# Estimating population ITN access at council level in Tanzania

**DOI:** 10.1186/s12936-022-04432-y

**Published:** 2023-01-05

**Authors:** Hannah Koenker, Matt Worges, Joshua Yukich, Peter Gitanya, Frank Chacky, Samwel Lazaro, Charles Dismas Mwalimu, Sijenunu Aaron, Raya Ibrahim, Faiza Abbas, Mwinyi Khamis, Deodatus Mwingizi, David Dadi, Ato Selby, Naomi Serbantez, Lulu Msangi, Dana Loll, Benjamin Kamala

**Affiliations:** 1 USAID Tanzania Vector Control Activity, Tropical Health, Baltimore, MD USA; 2 USAID Tanzania Vector Control Activity, Tropical Health, New Orleans, LA USA; 3 Tulane University, New Orleans, LA USA; 4 National Malaria Control Programme, Ministry of Health, Dodoma, Tanzania; 5 Zanzibar Malaria Elimination Programme, Zanzibar, Tanzania; 6 USAID Tanzania Vector Control Activity, Johns Hopkins University School of Public Health Center for Communication Programs, Dar Es Salaam, Tanzania; 7 U.S. President’s Malaria Initiative, Dar Es Salaam, Tanzania; 8 USAID Tanzania Vector Control Activity, Johns Hopkins University School of Public Health Center for Communication Programs, Baltimore, MD USA

**Keywords:** ITN, Malaria control, Vector control, Insecticide-treated net, Bed net, Tanzania, Zanzibar, Quantification, Sub-regional, School, Community-based, Distribution

## Abstract

**Background:**

Since 2013, the National Malaria Control Programme in mainland Tanzania and the Zanzibar Malaria Elimination Programme have implemented mass insecticide-treated net (ITN) distribution campaigns, routine ITN distribution to pregnant women and infants, and continuous distribution through primary schools (mainland) and community leaders (Zanzibar) to further malaria control efforts. Mass campaigns are triggered when ITN access falls below 40%. In this context, there is a need to monitor ITN access annually to assess whether it is below threshold and inform quantification of ITNs for the following year. Annual estimates of access are needed at the council level to inform programmatic decision-making.

**Methods:**

An age-structured stock and flow model was used to predict annual net crops from council-level distribution data in Tanzania from 2012 to 2020 parameterized with a Tanzania-specific net median lifespan of 2.15 years. Annual nets-per-capita (NPC) was calculated by dividing each annual net crop by mid-year council projected population. A previously fit nonparametric conditional quantile function for the proportion of the population with access to an ITN (ITN access) as a function of NPC was used to predict ITN access at the council level based on the predicted NPC value. These estimates were compared to regional-level ITN access from large household surveys.

**Results:**

For regions with the same ITN strategy for all councils, predicted council-level ITN access was consistent with regional-level survey data for 79% of councils. Regions where ITN strategy varied by council had regional estimates of ITN access that diverged from the council-specific estimates. Predicted ITN access reached 60% only when “nets issued as a percentage of the council population” (NPP) exceeded 15%, and approached 80% ITN access when NPP was at or above 20%.

**Conclusion:**

Modelling ITN access with country-specific net decay rates, council-level population, and ITN distribution data is a promising approach to monitor ITN coverage sub-regionally and between household surveys in Tanzania and beyond.

**Supplementary Information:**

The online version contains supplementary material available at 10.1186/s12936-022-04432-y.

## Background

Malaria is a significant public health burden in Tanzania, with 6 million cases in 2020 [1]. Insecticide-treated nets (ITNs) have been the cornerstone of malaria prevention since 2004 and have contributed to declines in malaria incidence from 162 per 1000 population in 2015 to 106 in 2020 [2]. Mass ITN distribution campaigns were implemented in mainland Tanzania in 2007–8, targeting children under five [3], and universal coverage campaigns were conducted nationwide in 2010–11 [4], in 23 of 26 mainland regions in 2015, and in 50 targeted councils of 12 regions in 2020. As part of its Keep Up Strategy [5], the National Malaria Control Programme (NMCP) has implemented annual distributions of ITNs through mainland primary schools since 2013, known as the School Net Programme (SNP). SNP was implemented in 3 regions in 2013, 2014, and 2015, then scaled up to 7 regions in 2016, and to 14 of 26 mainland regions from 2017 onward [6]. ITNs are also delivered through RCH (reproductive and child health) to pregnant women at their first antenatal care clinics (ANC) attendance and to infants at first measles immunization visits, known as IVD (immunization and vaccine development programme).

In Zanzibar, a semiautonomous region of Tanzania comprised of two islands, Unguja and Pemba, malaria prevalence has remained below low for the past decade and the islands are considered a pre-elimination setting. Zanzibar Malaria Elimination Programme (ZAMEP) has a policy of universal coverage with ITNs and conducted mass campaigns in 2006, 2008, 2012, 2016, 2020–2021, and continuous distribution through community-based coupon system ran from 2014 to 2016 and from 2018 to 2021. ITNs are also provided at antenatal care clinics and measles immunization visits since 2014.

The mainland Tanzania ITN strategy aims to maintain ITN access, defined by Roll Back Malaria and the World health Organization (WHO) as the proportion of the population that has access to an ITN within their household, assuming each ITN can protect up to two people. It can be considered as the proportion of the population who could use an ITN. The strategy relies on annual school distributions and delivery to pregnant women and infants at health facilities, utilizing targeted mass campaigns only when ITN access falls below 40%, to quickly scale back up to target levels of 80%. ITN access levels above 50% but below the target of 80% would reflect a need for additional ITNs to be distributed during the SNP; operationally this is managed by increasing the number of primary school classes receiving ITNs from previous years. Zanzibar’s community ITN distribution strategy likewise triggers mass replacement campaigns under a threshold of 50%. Thus, data on ITN access at the relevant operational level are needed to inform decisions on both the type of ITN distribution (catch-up campaign or continued SNP or community distribution) as well as the quantities of ITNs needed the subsequent year to maintain access at target levels.

The NMCP has stratified its malaria control interventions at the council (sub-regional) level, necessitating council-level estimates of ITN access to inform ITN quantification for programmatic decision-making. Large household surveys, such as the Demographic and Health Survey (DHS) or Malaria Indicator Survey (MIS), are implemented only every three to five years, and are representative at the regional level, with data available six months to a year after fieldwork, making these impractical for annual monitoring of ITN access. NMCP implements a school parasitaemia survey every two years to collect council-level parasitaemia data, but ITN access is collected at regional level. ZAMEP also relies on DHS and MIS data to provide periodic estimates of ITN coverage. Mobile phone surveys have been deployed with success in Tanzania (Worges et al. pers. commun.), but are not yet able to collect sufficient responses to stratify results below the regional level. The cost and resource implications of annual rapid in-person surveys are likewise daunting.

Given these objectives and associated challenges, the USAID Tanzania Vector Control Activity (TVCA) developed a modelling approach based on a stock and flow model used previously for ITN quantification [7] and as the basis of the NetCALC tool, used in Tanzania [5] and across sub-Saharan Africa to inform strategic planning for continuous distribution of ITNs. Net median lifespan parameters used Tanzania-specific estimates published in Bertozzi-Villa et al. [8]. This paper describes the approach to generating council-level estimates of ITN access and discusses the benefits and limitations of this modelling approach, with implications for future work.

## Methods

### Council-level estimates of ITN access

**Fig. 1:**
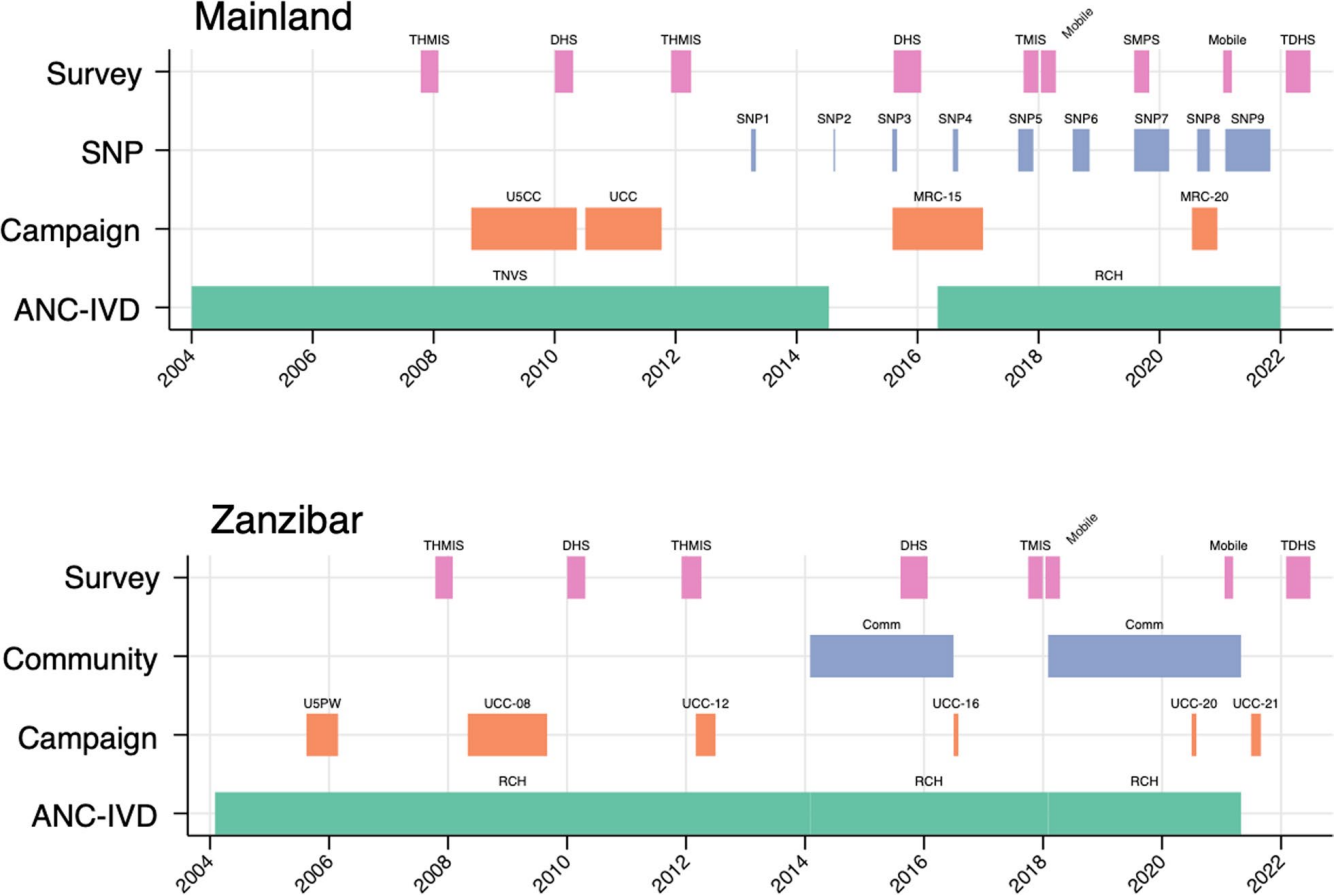
Timeline of ITN distributions and ITN coverage surveys in mainland and Zanzibar [3–6, 10–13] U5CC under-5 coverage campaign; *MRC* mass replacement campaign, *TNVS* Tanzania national voucher scheme, *SNP* school net programme, *RCH* reproductive and child health (antenatal care and/or immunization and vaccines), *DHS* demographic and health survey, *TMIS* Tanzania malaria indicator survey, *SMPS* school malaria and parasitaemia survey

ITN distribution data at the council level was provided by NMCP for each distribution channel in mainland Tanzania since 2004, summarized in Fig. 1. This included the Tanzania National Voucher Scheme (TNVS) from 2004 to 2014, and RCH (ANC and IVD) data from 2016 to present. Mass distributions included the under-five catch up campaign (U5CC) of 2007–8, the universal coverage campaign (UCC) of 2010–11, the 2015 mass replacement campaign (MRC), and the 2020 MRC. For the U5CC and the UCC, data was available only at the regional level; ITNs were allocated for this analysis across councils proportionally based on the council’s share of the regional population. SNP data was provided from 2013 to present. The National Bureau of Statistics provided mid-year council population projections for 2012–2030 from the 2012 Tanzania census data [9]. ITN distribution data for Zanzibar were provided by ZAMEP at the district level for the 2012 and 2016 universal coverage campaigns, community distribution, health facility distribution, and 2020–2021 mass campaign. Distributions were aggregated at an annual level for analysis.

**Fig. 2:**
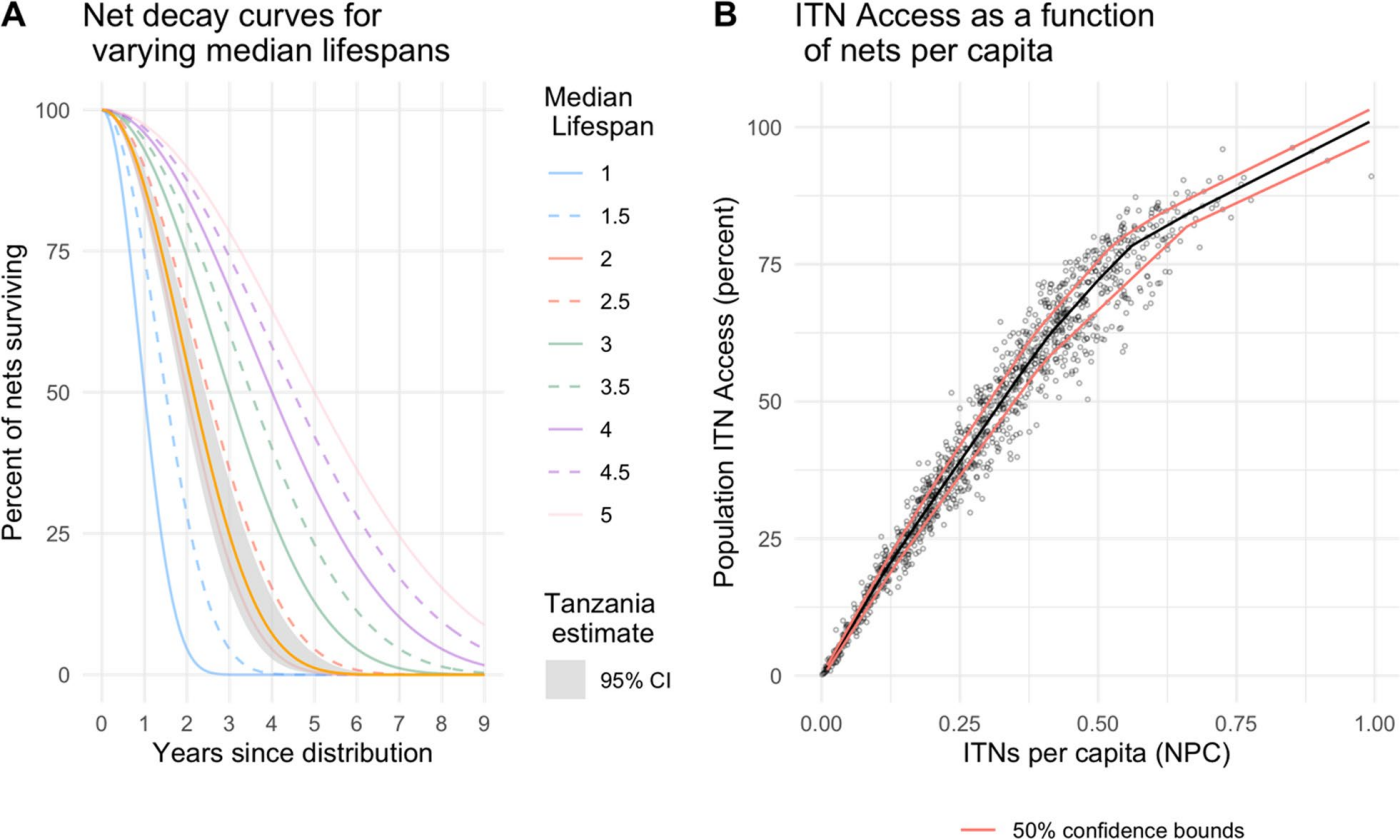
**A** Net survival decay curves for varying median lifespans, with Tanzania's estimated median lifespan shown in orange with its 95% CI. **B** Population ITN access vs ITNs per capita from 124 Demographic and Health Surveys and Malaria Indicator Surveys. Black line indicates the nonparametric conditional quantile fit, with red lines indicating the 50% confidence bounds

The analysis was restricted to 2012 onwards due to the availability of population data at council level. For each year, the stock and flow model used an estimated Tanzania specific median lifespan of 2.15 (95% CI 1.88–2.43) from Bertozzi-Villa et al. [8] to decay each crop of distributed nets annually. The net decay functions rely on smooth-compact loss function developed by Nakul Chitnis and described in Koenker et al. and Bhatt et al. [5, 14], and are shown in Fig. 2 Panel A; the formula is available as Additional file 1.

The total predicted net crop (consisting of all surviving nets from various channels to date) was summed for each year and council. This was then divided by the population projected to calculate nets-per-capita (NPC) in each year and council. To predict ITN access from NPC, a nonparametric conditional quantile function for ITN access as a function of NPC was fit to data from 124 Demographic Health Survey data and Malaria Indicator Surveys (MIS) using the “quantreg” package in R (v5.93; Koenker 2022, [15, 16]). Uncertainty was propagated using sensitivity analysis to establish best- and worst-case estimates of ITN access given the 95% confidence intervals for estimated median lifespan and 50% intervals for the function of ITN access vs NPC, shown in Fig. 2 Panel B.

**Fig. 3:**
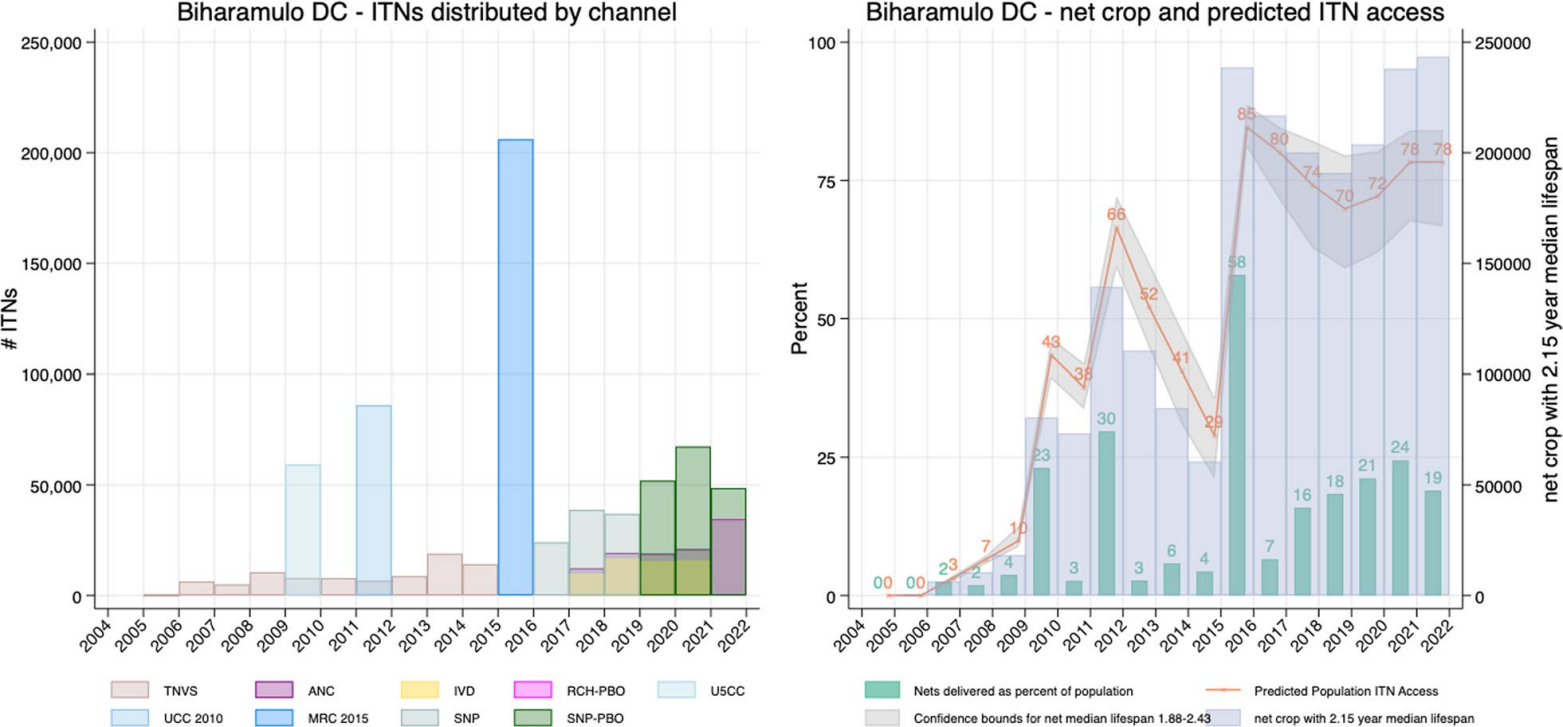
Example approach for estimating net crop and population ITN access using data from Biharamulo District Council. **a** ITN inputs from each distribution channel (note that columns representing different channels are overlaid); **b** total annual ITN inputs from all channels (green bars); ITN crop, having applied the decay function for a 2.15-year median lifespan to each annual input of ITNs (blue bars); population ITN access estimate (orange line) and its 95% confidence interval

To better visualize how the quantities of distributed ITNs relate to council population sizes, the number of ITNs distributed across all channels in a given year was divided by the council population, giving “nets issued as a percentage of the council population” (NPP). Thus, while NPC reflects total net crop per capita, NPP reflects annual inputs of nets relative to population. NPP is helpful particularly as ANC and IVD channels were reinstated in mainland in 2016–17, and as the number of SNP targeted classes has varied over time and by council, as illustrated in Fig. 3.

### Survey data on population ITN access

Public population-based household survey datasets for 2007–8 Tanzania HIV/AIDS and Malaria Indicator Survey (THMIS), 2010 DHS, 2011–12 THMIS, 2015–16 DHS/MIS, and 2017 MIS were obtained with permission from dhsprogram.org, and population ITN access for each region was calculated following Roll Back Malaria guidelines [17]. ITN access estimates at regional level from the 2019 SMPS were obtained from NMCP. Net access estimates at regional level from mobile phone surveys conducted in 2018 (23 mainland regions) and in 2021 (26 mainland regions) were extracted from project reports. As the two mobile phone surveys relied on self-report rather than observation of nets in the household, ITN status of the nets could not be confirmed. The majority of nets in Tanzanian households are ITNs; untreated nets comprised less than 10% of nets in the 2017 TMIS [18].

## Results

### Predictions of ITN access at council level

**Fig. 4:**
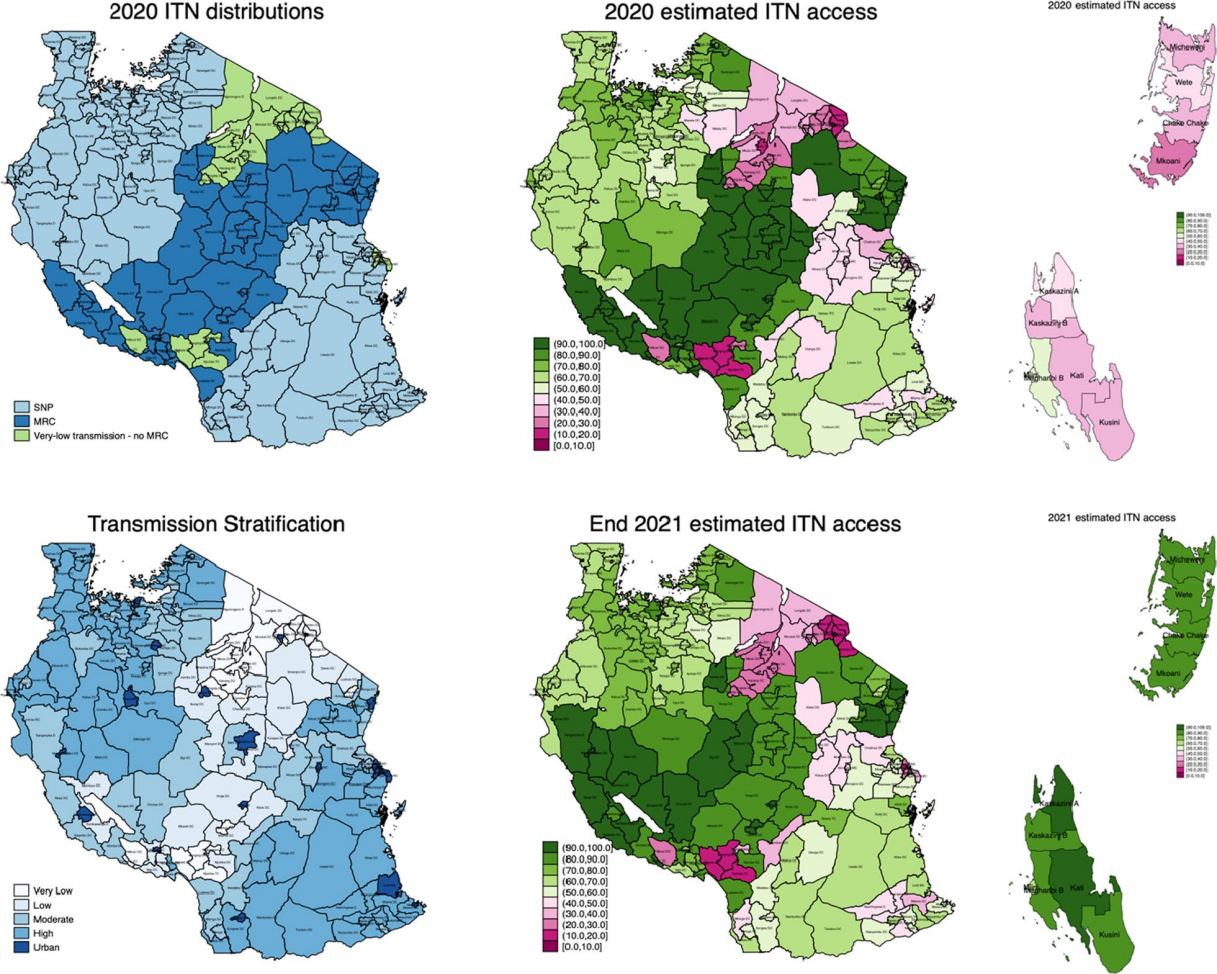
Council-level classification of ITN strategy (top left) and the council-level stratification by transmission intensity (bottom left). Map of estimates of ITN access for 2020–2021 mainland (top middle; bottom middle) and for 2020–2021 Zanzibar (top right; bottom right)

ITN access predictions for 2020 and 2021 ranged from 16.7% to 100% across 184 councils of mainland Tanzania and are summarized in Fig. 4. Annual nets-per-capita (NPC) ranged from 0.10 to 1.39. NPC and estimated ITN access were highest in the councils that had received ITNs in the 2020 MRC, and lowest in the very-low transmission councils that received neither the 2020 MRC nor were targeted for SNP. Of the 29 councils with estimated ITN access below 40% at the end of 2020, 22 were councils classified as very-low-transmission, one was moderate transmission (Chalinze DC in Pwani), and six were urban (Arusha, Singida, and four of Dar es Salaam’s five councils [19]; however, all six of these urban councils are considered by NMCP to have very-low transmission. Overall for 2020, 27.0% of councils (n = 50) had access estimates between 80 and 100%, 48.7% (n = 90) were between 50 and 79%, 8.1% (n = 15) were between 40 and 50%, and 16.2% (n = 30) were less than 40%. For 2021, 27.6% of councils (n = 51) had access above 80%, 49.7% (n = 92) were between 50 and 79%, 5.4% (n = 10) were between 40 and 50%, and 17.3% (n = 32) were under 40%.

In Zanzibar, ITN access estimates for 2020 ranged from 28.9 to 65.4% across 11 districts, and NPC ranged from 0.18 to 0.44. No districts had ITN access greater than 80%; three districts were between 50 and 79%, two districts were between 40 and 50%, and the remaining six districts were less than 40%. Following Zanzibar’s 2021 mass campaign, ten of Zanzibar’s eleven districts had an estimated ITN access of more than 80%.

**Fig. 5:**
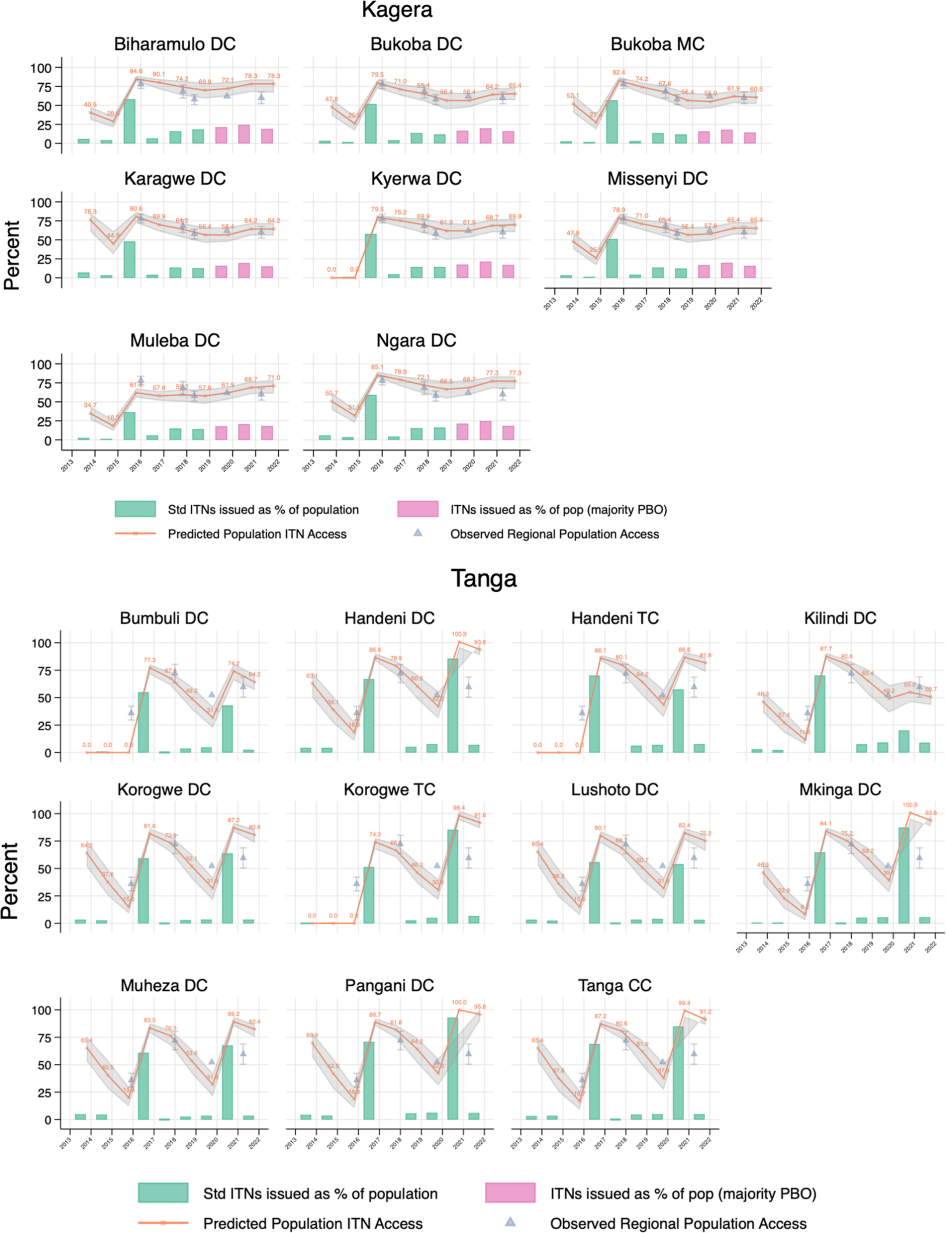

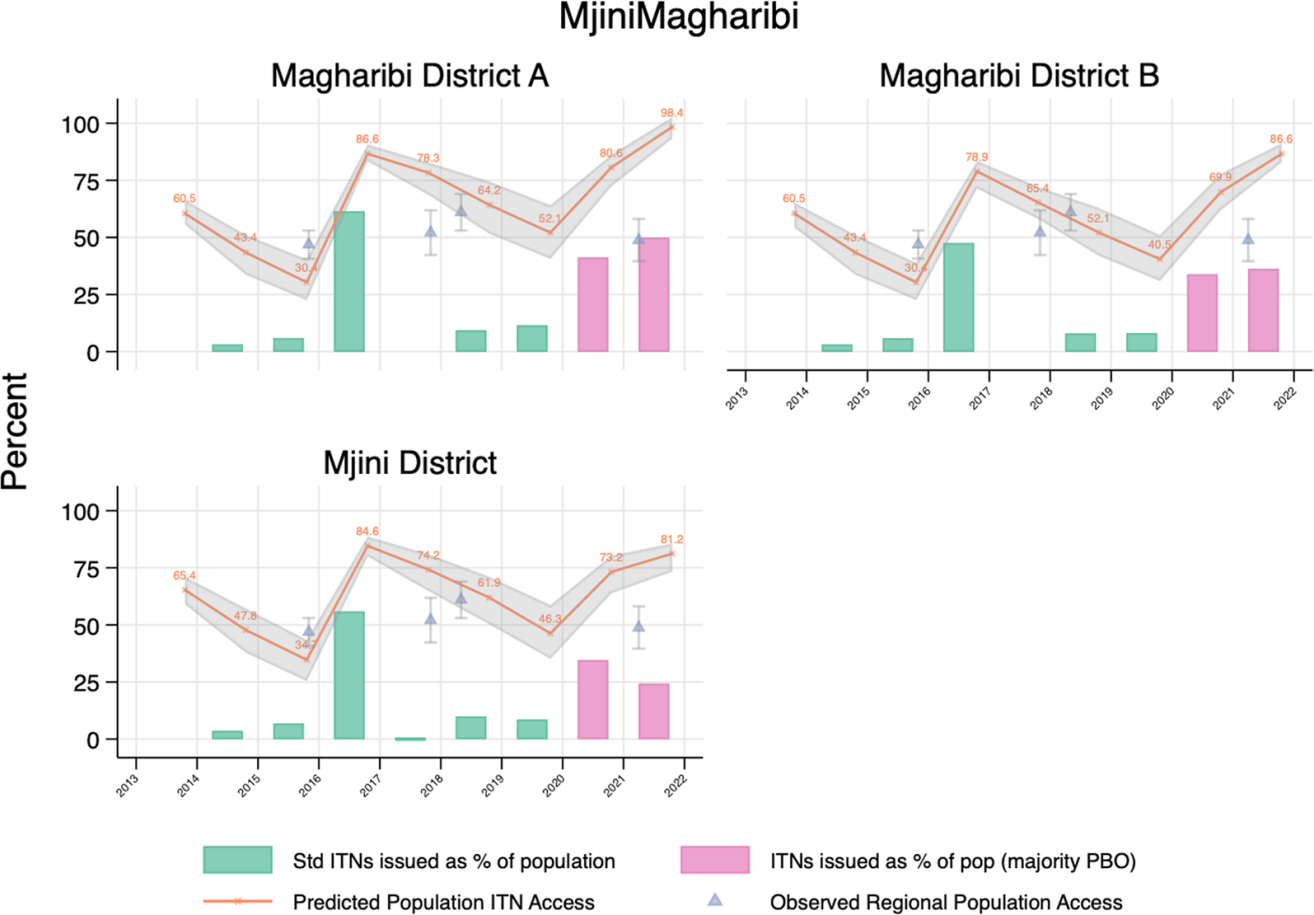
Estimated population ITN access (orange line), ITNs delivered as percent of the population (NPP—green and pink bars), and survey estimates of population ITN/net access (triangles) for districts within the regions of Kagera, Tanga, and Mjini Magharibi (Zanzibar)

Figure 5 provides selected examples of (a) the council level population ITN access estimates (orange line), (b) ITNs delivered as percent of the population (NPP—green/pink bars), and (c) survey estimates of population ITN/net access (triangles) for districts within Kagera, Tanga, and Mjini Magharibi regions. For Kagera, the 2015 mass replacement campaign is evident in the green line’s spike, and the fluctuation in ITNs delivered under SNP + RCH. For Tanga, the 2015 and 2020 campaigns are likewise evident. In Mjini Magharibi, the initial access is high due to the 2012 mass campaign, followed by the 2016 mass campaign, and the 2020–2021 mass campaign. Community distribution occurred to a limited degree between campaigns. The complete set of graphs for all councils in Tanzania and districts in Zanzibar is included as Additional file 2.

Confidence intervals (CIs) for modelled estimates of council-level population ITN access in mainland overlapped those of regional-level observed ITN access from the 2017 Tanzania MIS and 2021 mobile phone survey in 79.1% of councils targeted for school distribution. Within regions not targeted for school distribution, CIs overlapped in only 44.1% of councils. In Zanzibar, modelled and observed estimates overlapped in 42.4% of districts (Additional file 2).

### Scale-up of RCH distribution

**Fig. 6:**
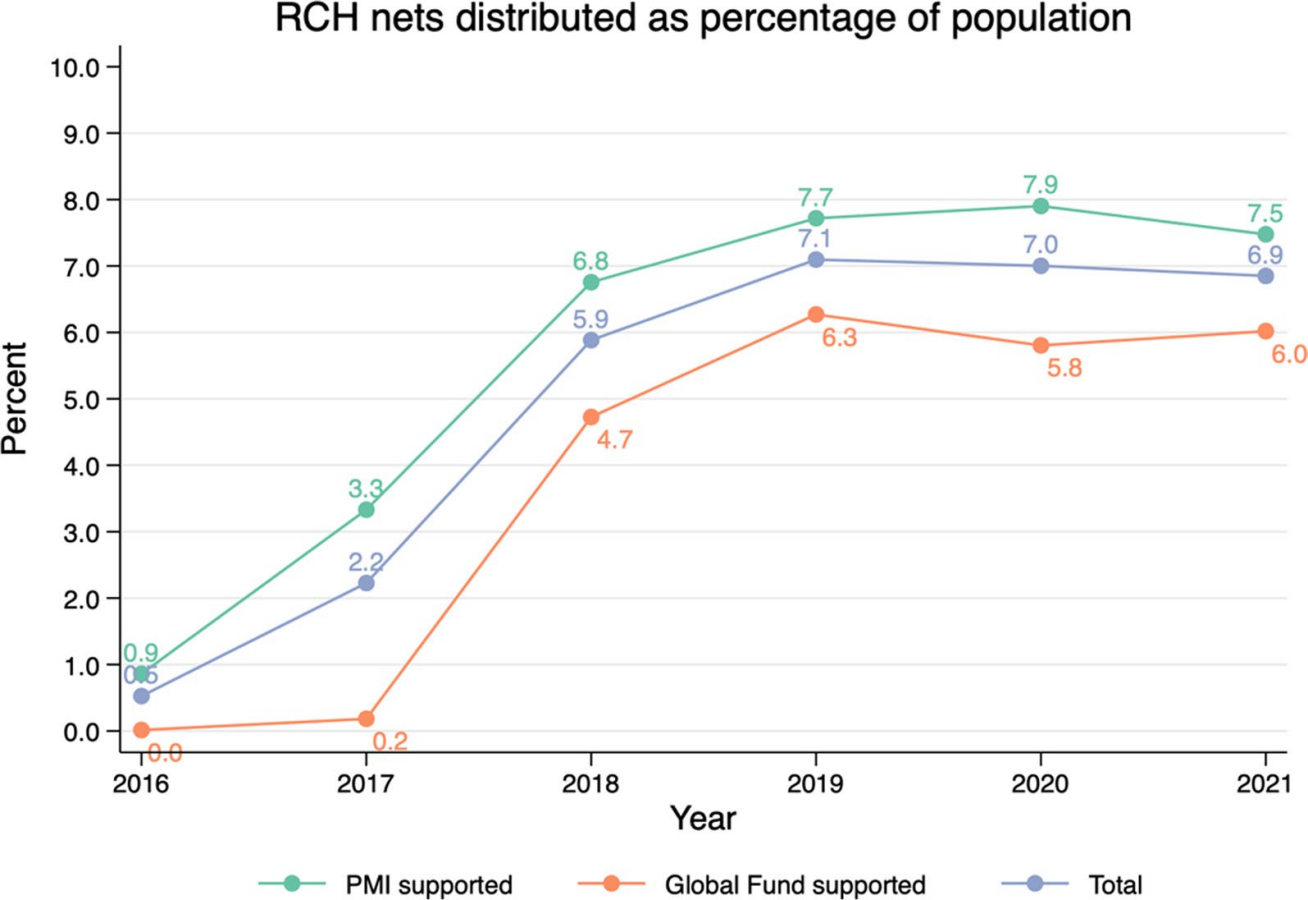
ITNs distributed to pregnant women and infants, 2016–2021, as a percent of the total population

RCH distribution targets pregnant women, typically assumed to represent 5% of the population, and children under 1 year of age, estimated at 3.2% of the Tanzanian population for 2022 [20]. A highly effective RCH programme could thus expect to provide nets per capita of 8% yearly if it reaches all targeted individuals. RCH nets distributed per capita since free RCH commenced in 2016 is shown in Fig. 6, by year and by donor. As RCH deliveries scaled up in 2016–2017, RCH nets per capita increased, reaching nearly 8% in PMI-supported regions and around 6% in Global Fund supported regions over 2019–2021.

### Quantification of ITNs for annual school or community distribution

**Fig. 7:**
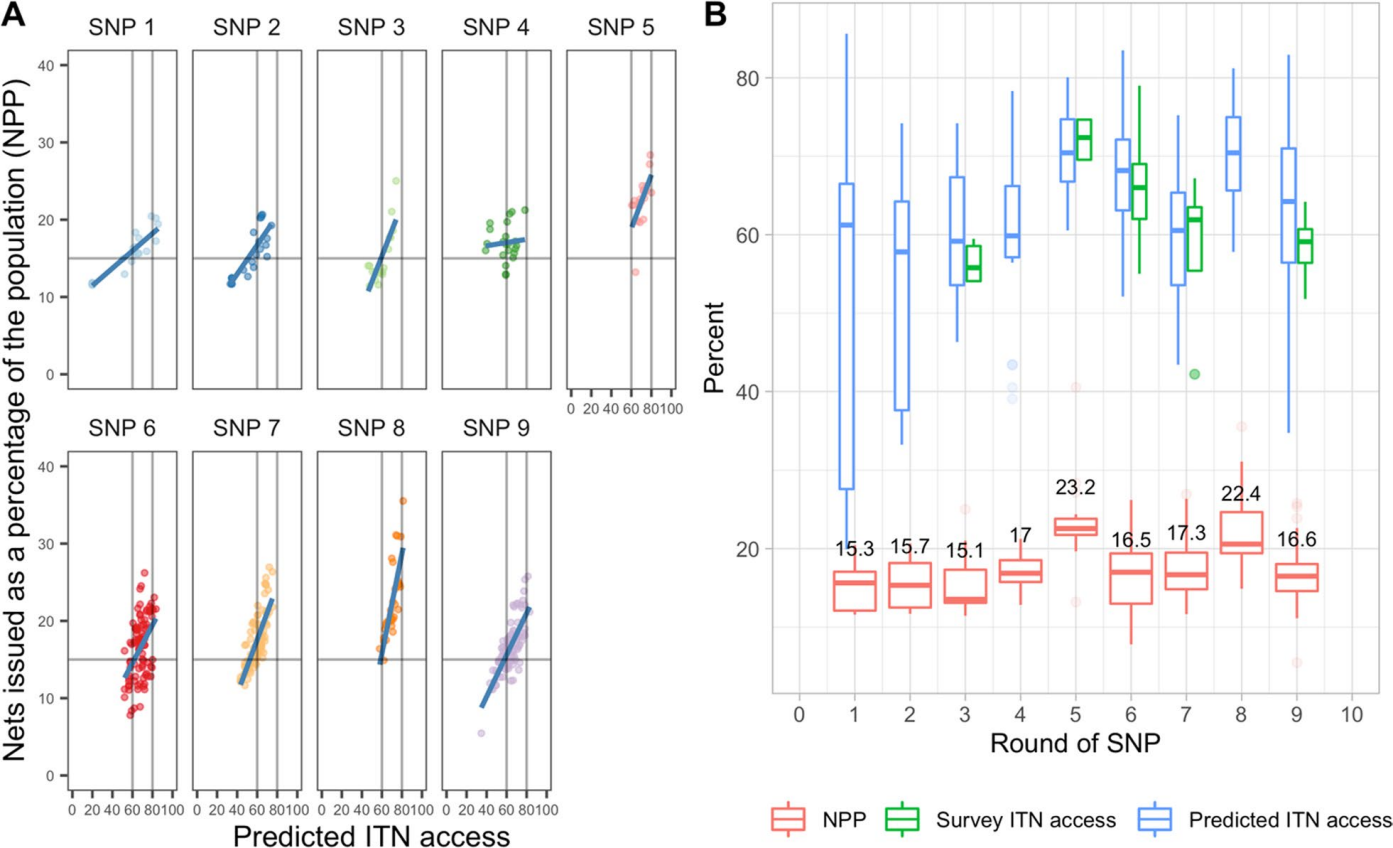
**A** ITNs issued in SNP and RCH as a percent of the council population (NPP) vs predicted ITN access. Dots represent council-years, restricted to 416 council-years at least three years out from the previous mass campaign. Nineteen districts with missing population/distribution data are excluded. Linear fit lines are shown in blue. **B** Boxplot showing mean ITNs issued as a percent of population (NPP) in red, along with predicted ITN access (blue), and ITN access observed in household surveys (green), showing correspondence of predicted and observed results, and increases in ITN access in years when NPP exceeds 20

To facilitate quantification of school and community distributions on an annual basis, it would be useful to have a simple multiplier based on population, in the way that “population/1.8” is used to quantify ITNs for mass campaigns [21]. Nets distributed as a percentage of the population (NPP) can be used for this purpose. For mass campaigns in Tanzania, NPP averaged 66% with a median of 61.4% for the 2015 mainland MRC, and averaged 69.3% with a median of 68.9% for the 2020 MRC. The Zanzibar 2016 MRC averaged 48.6% NPP with a median of 52.6%, and in 2021 this was 51.8% and 51.7%, respectively (NPP of 55.5% corresponds to “population / 1.8”). For continuous and routine distribution on mainland, NPP varied over time depending on quantification approaches as well as funding availability for ITN procurement. Figure 7 below plots NPP against predicted ITN access, excluding 19 outlier councils where population estimates and ITN distribution data are out of alignment due to district boundary changes and/or classification issues (Additional file 3), and is restricted to the 416 council-years in which the SNP had occurred at least three years since the council’s last mass campaign, representing a ‘steady state’ of continuous distribution. In 34% of these council-years, NPP for SNP was less than 15%, peaking in SNP5 at an average of 23.2 (Fig. 7 Panel B) and corresponding with higher ITN access in the 2017–18 TMIS and with predicted ITN access levels.

Linear fit lines in Fig. 7 Panel A show that, with the exception of SNP6, predicted ITN access reaches 60% only when NPP from both SNP and RCH exceeds 15%, and approaches 80% ITN access when NPP from both channels is around 20%.

## Discussion

For regions that maintained a consistent ITN strategy across councils, the council-level estimates of population ITN access were reasonably well-aligned with the overall regional estimates of ITN access from observational studies. For councils having received multiple rounds of SNP, this method of estimating ITN access may serve to understand the likely levels of access in between large household surveys, such as the school malaria parasitaemia survey or Malaria Indicator Surveys/Demographic and Health Surveys.

In regions where ITN strategies vary across councils, one would expect modelled council estimates to diverge from the regional estimate. This is most apparent in regions where the 2020 Mass Replacement Campaign was not implemented in all councils. Councils targeted for the 2020 MRC would have higher modelled ITN access than the regional average; while very-low transmission councils that were not targeted for the 2020 campaign would have lower modelled ITN access than the regional average.

The estimates are likewise subject to additional limitations.

1. Shifting council boundaries/designations over time. For councils created since the 2012 census, population data is missing. Nineteen of the 184 councils are likely subject to this limitation, increasing the uncertainty of their estimates, with more details provided in Additional file 3.
2. Population shifts not accounted for in the 2012 census projections. While National Bureau of Statistics makes every effort to project population accounting for both growth and geographic shift/urbanization, the current council populations may be significantly different from their projected estimates.
3. Acquisition of ITNs outside of public sector distributions. The 2017 Tanzania MIS reported that Dar es Salaam, Mtwara, Lindi, and Pwani had substantial proportions of households that had purchased nets (27 to 53%). Modelled estimates of ITN access were substantially lower than regional observed estimates in Dar es Salaam in particular, possibly reflecting the prevalence of net purchases (both treated and untreated).
4. Variability in net median lifespan across the country. Estimates of net median lifespan used for this model were derived from national-level data. It is likely that net care behaviors, wear and tear factors, and retention behavior varies across the country, or may have changed over time, and this would impact estimates of ITN access. Median lifespans above 2.5 years are outside of the confidence bounds of the current estimates.
5. Oversaturation of ITNs in certain households. The SNP targets households with children attending primary school (around 50% of households in Tanzania), and encourages families to redistribute ITNs they do not need to other households. While some households do report doing this, it is not done at a sufficient level to ensure perfectly equitable ITN coverage to untargeted households [22]. During the MRC, smaller households are more likely to receive the needed number of nets, while larger households may miss out on their full complement of nets due to capping strategies meant to limit fraud and achieve equity when net gaps are identified [23]. It will be important for future work to assess whether the relationship between NPC and ITN access is different in areas receiving the SNP compared to areas implementing mass campaigns, as this will inform adjustments to the ITN strategy.
6. Difficulties in obtaining complete ITN distribution data. Tanzania has excellent data systems for capturing ITNs delivered and issued under the channels it currently operates. However, this method is less useful in areas where ITN records are incomplete or inconsistently reported. Decisions must be taken about whether to define ITN inputs as all ITNs delivered to a certain locality or only those issued; nets remaining after a mass campaign are frequently issued later on either through ad-hoc distributions or integrated into routine ITN channels; they do not simply disappear. The model is unlikely to be sensitive to these small details but nonetheless a consistent approach must be defined.

Despite these limitations, the present approach is a promising method for estimating ITN access for areas, particularly sub nationally, where it is too expensive, time-consuming, or logistically impossible to implement regular observational studies. The approach is best suited for countries or subnational areas with consistent ITN strategies, reliable population data, and complete ITN distribution records.

As more countries conduct stratifications and apply interventions targeted sub nationally, it will be imperative to be able to assess intervention coverage at more granular levels. This approach is likely to be useful in many settings where ITN access estimates are needed between large distributions to inform annual planning. It would also be useful when estimating ITN access for national strategic plans and Global Fund applications for countries whose DHS or MIS is several years old.

Analysis of the RCH channel indicates that PMI-supported regions are performing well, distributing ITNs at close to target levels of 8%. Regions supported with funding from Global Fund are slightly behind, delivering ITNs at around 6% of the population. Similarly, the historical levels of ITN inputs (nets distributed as a percentage of the population) and the resulting ITN coverage at both council and regional level indicate that ITN access is closer to target levels when the SNP quantification (independent of RCH) is equivalent to at least 15% of the target population. It is crucial to consider the contributions from both RCH and SNP together, and to plan for quantifications that account for performance disparities between zones and regions. SNP rounds that deliver quantities of ITNs equivalent to less than 15% of the population are not predicted to be sufficient to maintain ITN access near target levels of 80%.

## Conclusion

Modelling ITN access with country-specific net decay rates, and council-level population and ITN distribution data may be a promising approach to monitor ITN coverage at the sub-regional level and between household surveys both in Tanzania and beyond. However, more work is needed to improve estimates, fine tune ITN retention times at smaller scales, and to inform future quantification approaches.

## Supplementary Information

**Additional file 1.** Net decay formula.

**Additional file 2.** Graphs of estimated ITN access and ITNs issued from 2013–2022 for all councils in Tanzania.

**Additional file 3.** Table of councils with incomplete or missing ITN or population data.

## Data Availability

ITN delivery data are available upon request from the NMCP and ZAMEP. Population estimates are available upon request from the Tanzania National Bureau of Statistics.
