# Supplementary material for: Estimating population ITN access at council level in Tanzania
Source: Malar J. 2023 Jan 5;22:4. doi: 10.1186/s12936-022-04432-y (PMC9815063; doi:10.1186/s12936-022-04432-y)
Supplement: Supplementary file 1 — Additional file 1. Net decay formula. [file 12936_2022_4432_MOESM1_ESM.docx]

# **Additional file information**

Net decay formula:

Where *c_n_* = net crop in the starting year, *c_n+t_* = net crop in a subsequent year, k = the constant 20, *t* is the time in years since the net distribution, and *L* is the value 11.75, representing a median lifespan of 2.15 years.

$$c_{n+t}= c_{n}\times\exp\left( k-\frac{k}{1- \left( \frac{t}{L} \right)^{2}} \right)$$

Council level graphs are available as **Additional file 1.**

**Additional file 2:** Table of councils with incomplete or missing ITN or population data
