# Supplementary material for: Estimating population ITN access at council level in Tanzania
Source: Malar J. 2023 Jan 5;22:4. doi: 10.1186/s12936-022-04432-y (PMC9815063; doi:10.1186/s12936-022-04432-y)

Estimating population ITN access at council level in Tanzania: Additional File 1

Hannah Koenker, Tropical Health LLP

December 23, 2022

Global Fund Regions

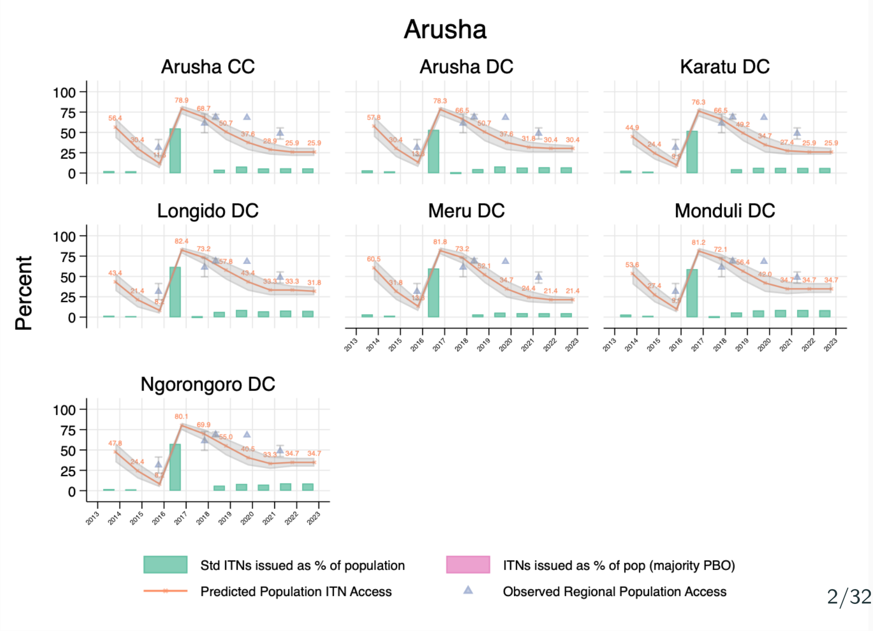
Arusha
Arusha CC
Arusha DC
Karatu DC
Longido DC
Meru DC
Monduli DC
Ngorongoro DC
Percent
Std ITNs issued as % of population
ITNs issued as % of pop (majority PBO)
Predicted Population ITN Access
Observed Regional Population Access

Dar es Salaam

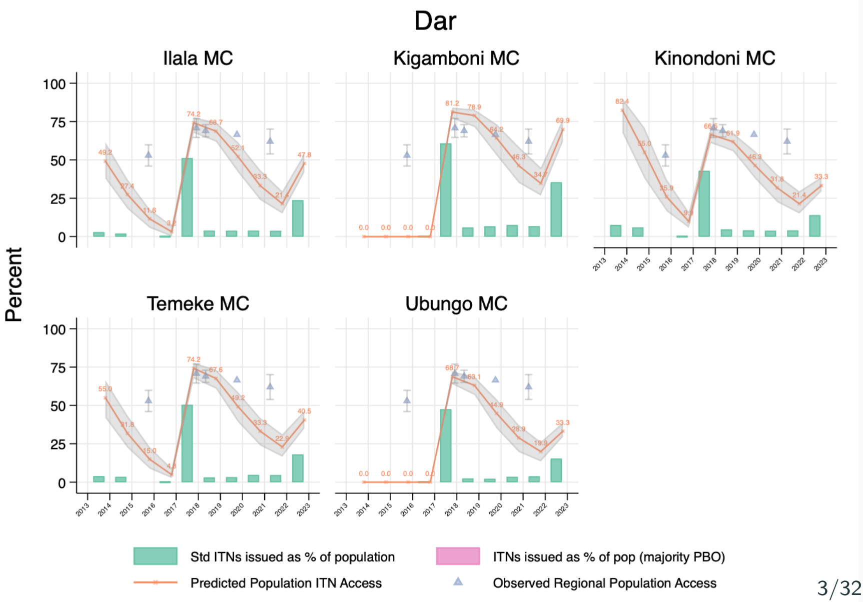

Dodoma

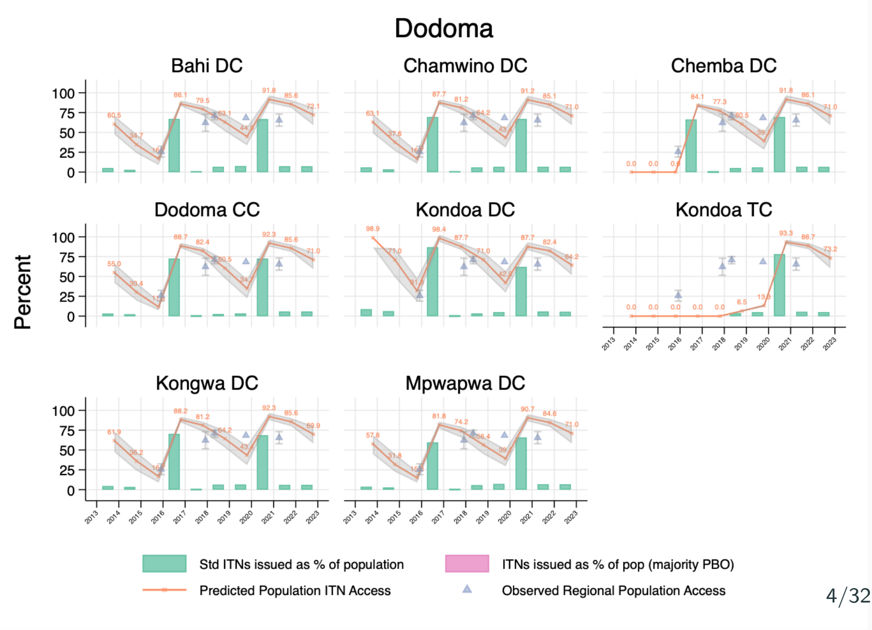
Dodoma
Bahi DC
Chamwino DC
Chemba DC
Dodoma CC
Kondoa DC
Kondoa TC
Kongwa DC
Mpwapwa DC
Percent
Std ITNs issued as % of population
ITNs issued as % of pop (majority PBO)
Predicted Population ITN Access
Observed Regional Population Access

Iringa

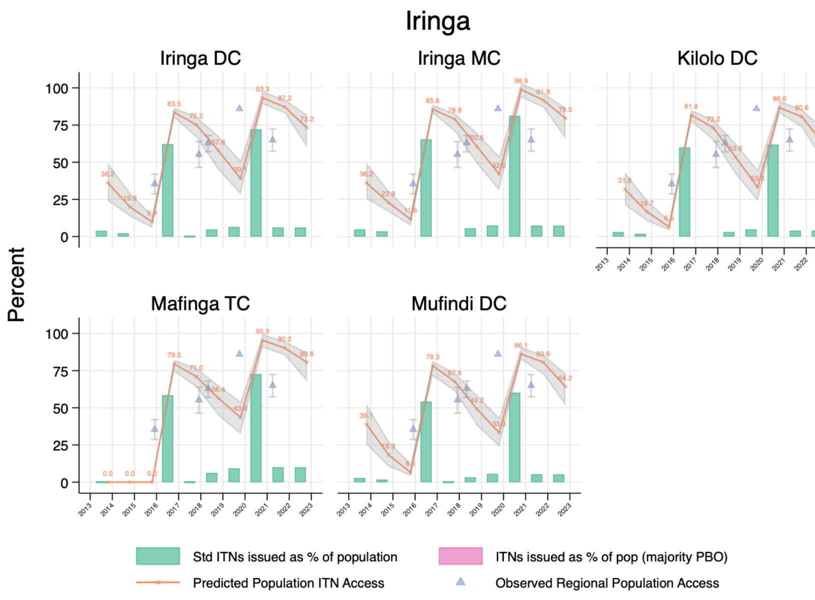

Iringa
Iringa DC
Iringa MC
Kilolo DC
Mafinga TC
Mufindi DC
Percent
100
75
50
25
0
2013
2014
2015
2016
2017
2018
2019
2020
2021
2022
2023
Std ITNs issued as % of population
ITNs issued as % of pop (majority PBO)
Predicted Population ITN Access
Observed Regional Population Access

Kilimanjaro

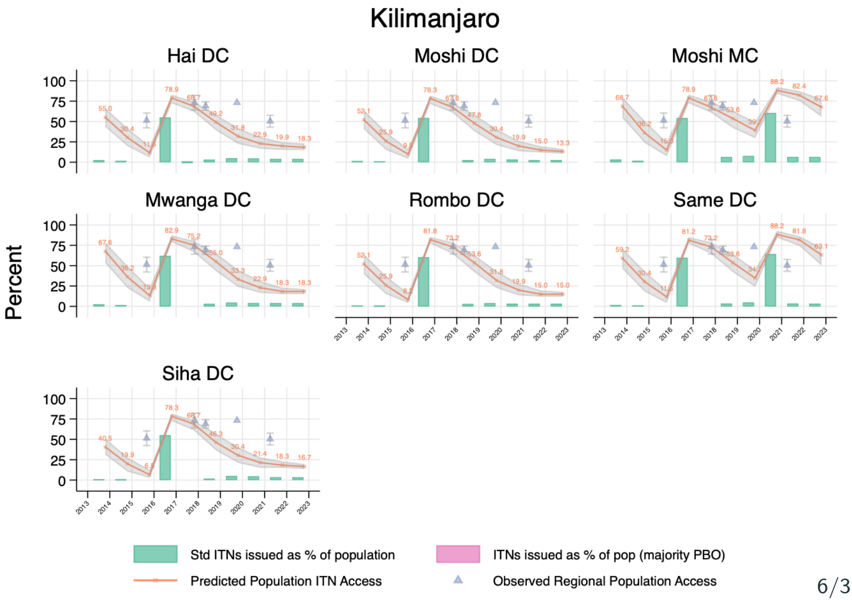

Manyara

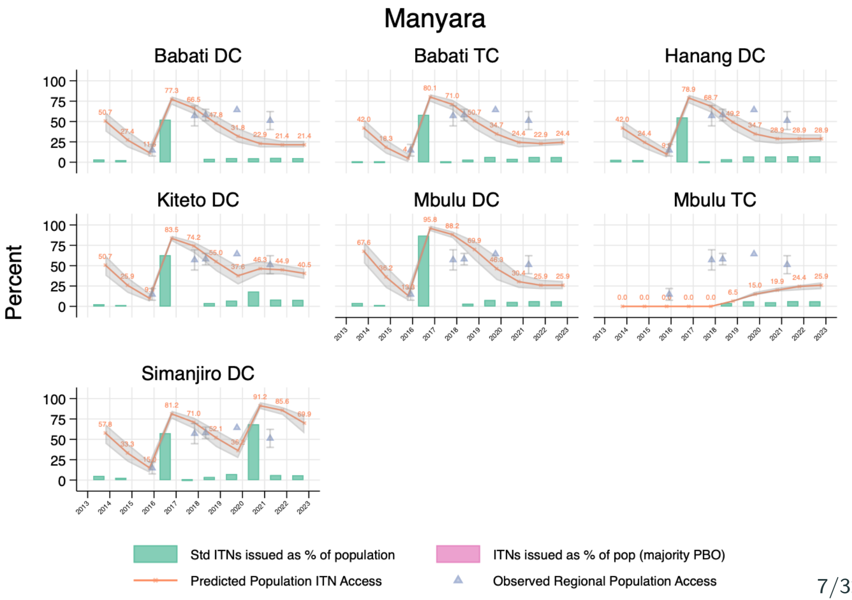

Manyara
Babati DC
Babati TC
Hanang DC
Kiteto DC
Mbulu DC
Mbulu TC
Simanjiro DC
Percent
Std ITNs issued as % of population
ITNs issued as % of pop (majority PBO)
Predicted Population ITN Access
Observed Regional Population Access

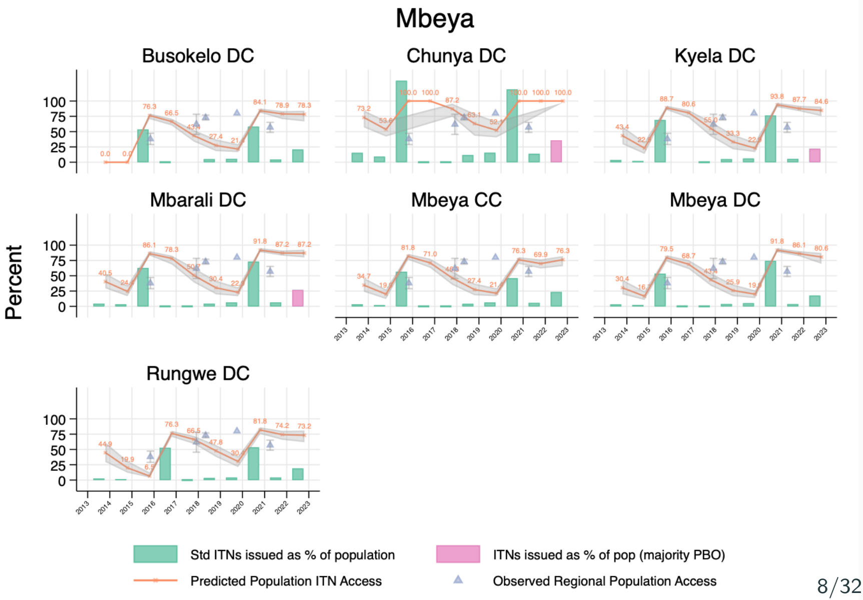
Mbeya
Busokelo DC
Chunya DC
Kyela DC
Mbarali DC
Mbeya CC
Mbeya DC
Rungwe DC
Percent
Std ITNs issued as % of population
ITNs issued as % of pop (majority PBO)
Predicted Population ITN Access
Observed Regional Population Access

Njombe

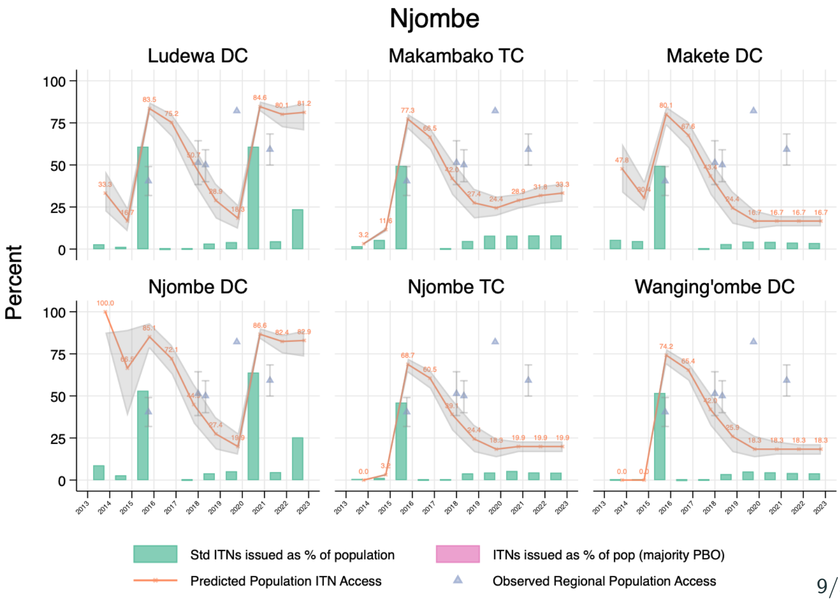

Njombe
Ludewa DC
Makambako TC
Makete DC
Njombe DC
Njombe TC
Wanging'ombe DC
Percent
0
25
50
75
100
2013
2014
2015
2016
2017
2018
2019
2020
2021
2022
2023
Std ITNs issued as % of population
ITNs issued as % of pop (majority PBO)
Predicted Population ITN Access
Observed Regional Population Access

Rukwa

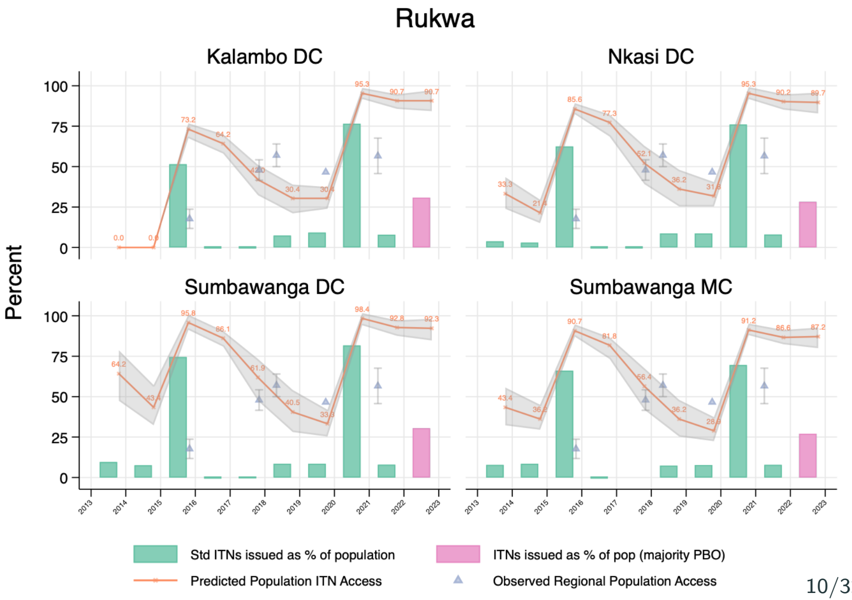

Rukwa
Kalambo DC
Nkasi DC
Sumbawanga DC
Sumbawanga MC
Percent
Std ITNs issued as % of population
ITNs issued as % of pop (majority PBO)
Predicted Population ITN Access
Observed Regional Population Access

Singida

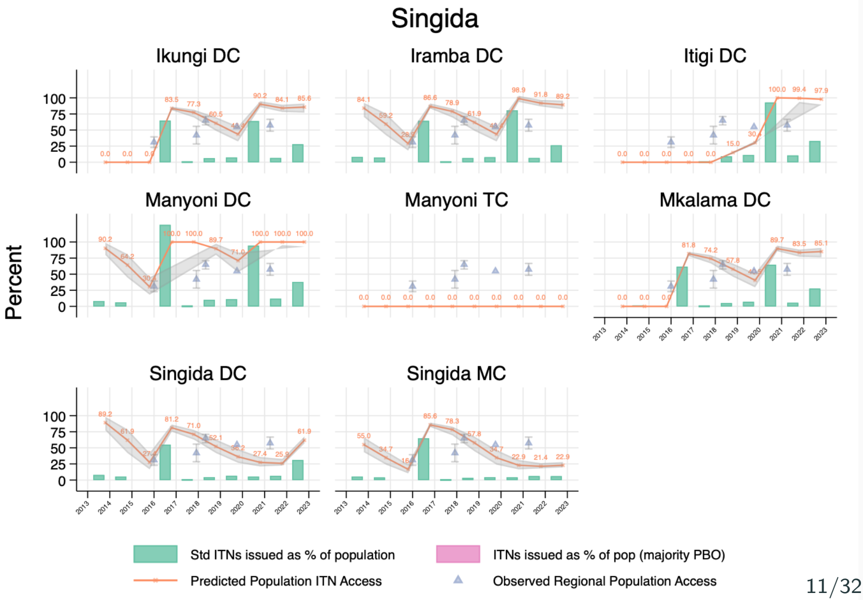
Singida
Ikungi DC
Iramba DC
Itigi DC
Manyoni DC
Manyoni TC
Mkalama DC
Singida DC
Singida MC
Percent
Std ITNs issued as % of population
ITNs issued as % of pop (majority PBO)
Predicted Population ITN Access
Observed Regional Population Access

Songwe

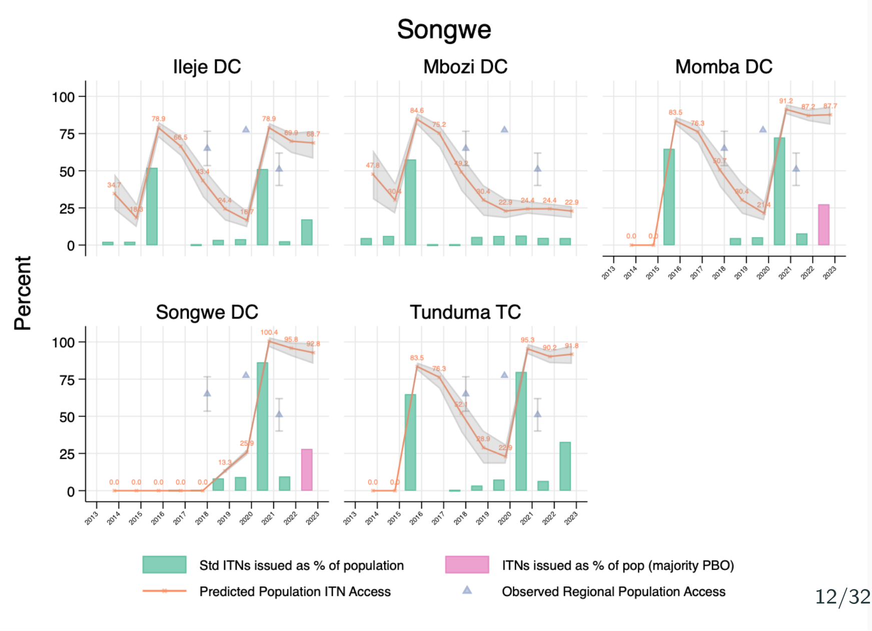

Songwe
Ileje DC
Mbozi DC
Momba DC
Songwe DC
Tunduma TC
Percent
Std ITNs issued as % of population
ITNs issued as % of pop (majority PBO)
Predicted Population ITN Access
Observed Regional Population Access

Tanga

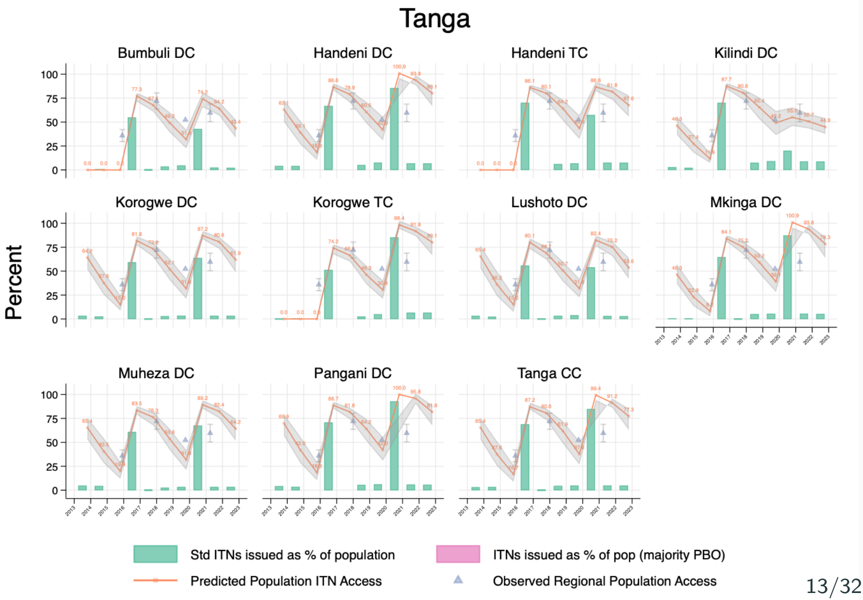
Tanga
Bumbuli DC
Handeni DC
Handeni TC
Kilindi DC
Korogwe DC
Korogwe TC
Lushoto DC
Mkinga DC
Muheza DC
Pangani DC
Tanga CC
Percent
Std ITNs issued as % of population
ITNs issued as % of pop (majority PBO)
Predicted Population ITN Access
Observed Regional Population Access

PMI Regions

Geita

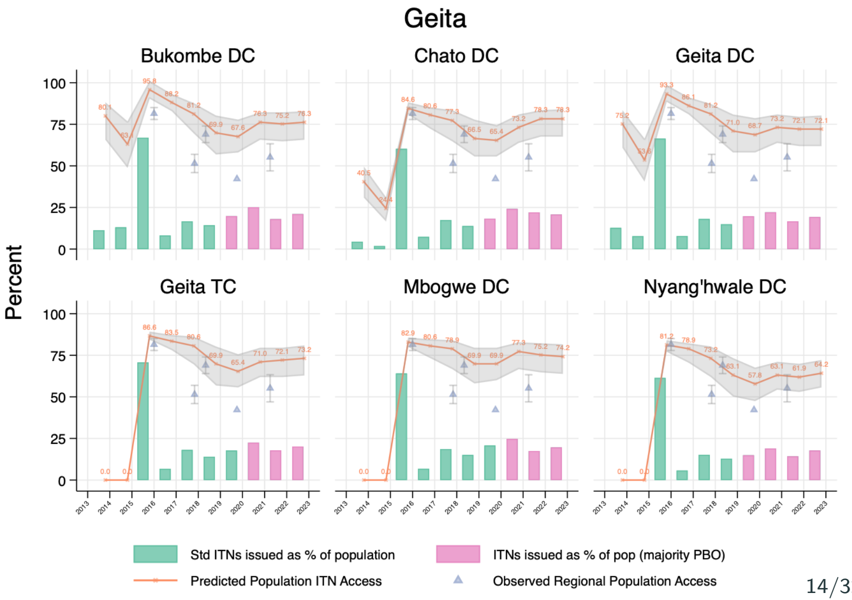
Geita
Bukombe DC
Chato DC
Geita DC
Geita TC
Mbogwe DC
Nyang'hwale DC
Percent
Std ITNs issued as % of population
ITNs issued as % of pop (majority PBO)
Predicted Population ITN Access
Observed Regional Population Access

Kagera

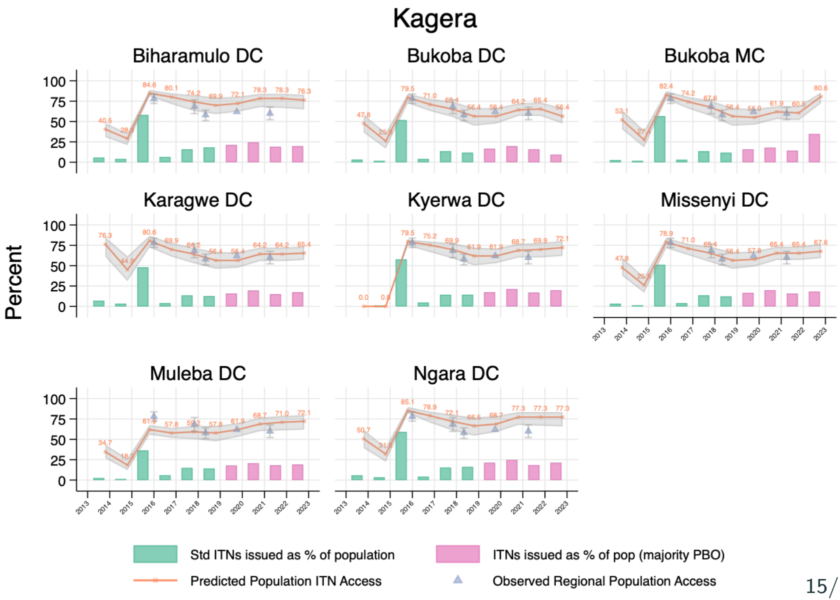

Kagera
Biharamulo DC
Bukoba DC
Bukoba MC
Karagwe DC
Kyerwa DC
Missenyi DC
Muleba DC
Ngara DC
Percent
100
75
50
25
0
2013
2014
2015
2016
2017
2018
2019
2020
2021
2022
2023
Std ITNs issued as % of population
ITNs issued as % of pop (majority PBO)
Predicted Population ITN Access
Observed Regional Population Access

Katavi

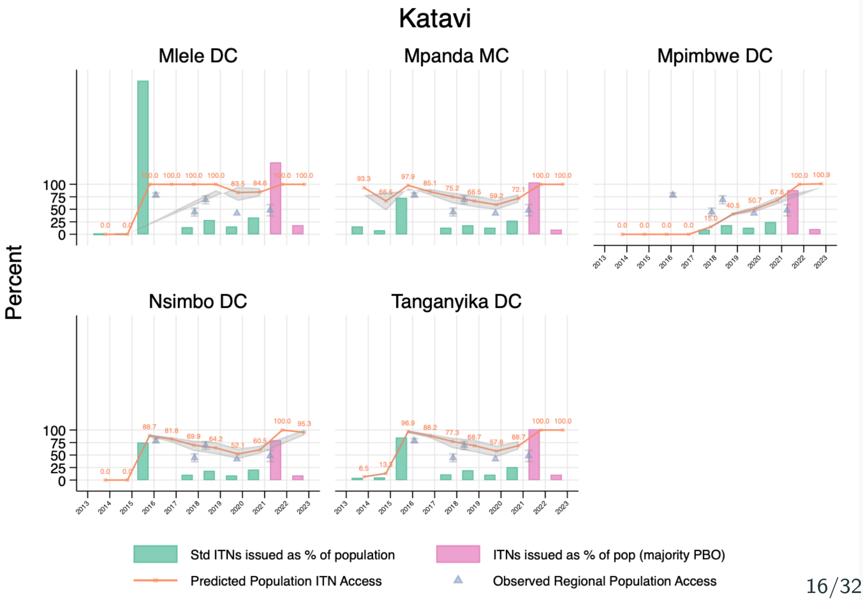
Katavi
Mlele DC
Mpanda MC
Mpimbwe DC
Nsimbo DC
Tanganyika DC
Percent
Std ITNs issued as % of population
ITNs issued as % of pop (majority PBO)
Predicted Population ITN Access
Observed Regional Population Access

Kigoma

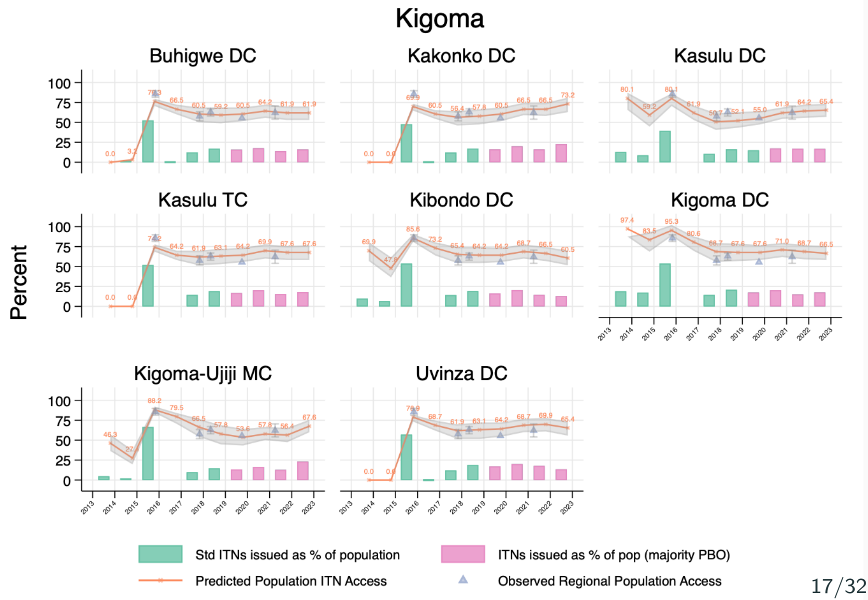

Kigoma
Buhigwe DC
Kakonko DC
Kasulu DC
Kasulu TC
Kibondo DC
Kigoma DC
Kigoma-Ujiji MC
Uvinza DC
Percent
Std ITNs issued as % of population
ITNs issued as % of pop (majority PBO)
Predicted Population ITN Access
Observed Regional Population Access

Lindi

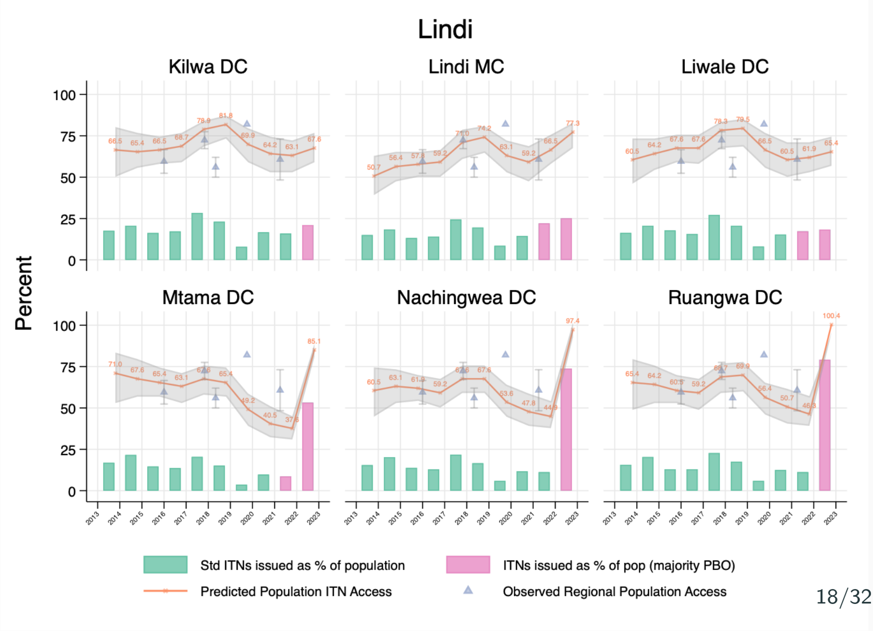

Lindi
Kilwa DC
Lindi MC
Liwale DC
Mtama DC
Nachingwea DC
Ruangwa DC
Percent
100
75
50
25
0
2013
2014
2015
2016
2017
2018
2019
2020
2021
2022
2023
66.5 65.4 66.5 68.7 78.9 81.8 69.9 64.2 63.1 67.6
50.7 56.4 57.8 59.2 71.0 74.2 63.1 59.2 66.5 77.3
60.5 64.2 67.6 67.6 78.3 79.5 66.5 60.5 61.9 65.4
71.0 67.6 65.4 63.1 67.6 65.4 49.2 40.5 37.6 85.1
60.5 63.1 61.9 59.2 67.6 67.6 53.6 47.8 44.9 97.4
65.4 64.2 60.5 59.2 68.7 69.9 56.4 50.7 46.3 100.4
Std ITNs issued as % of population
ITNs issued as % of pop (majority PBO)
Predicted Population ITN Access
Observed Regional Population Access

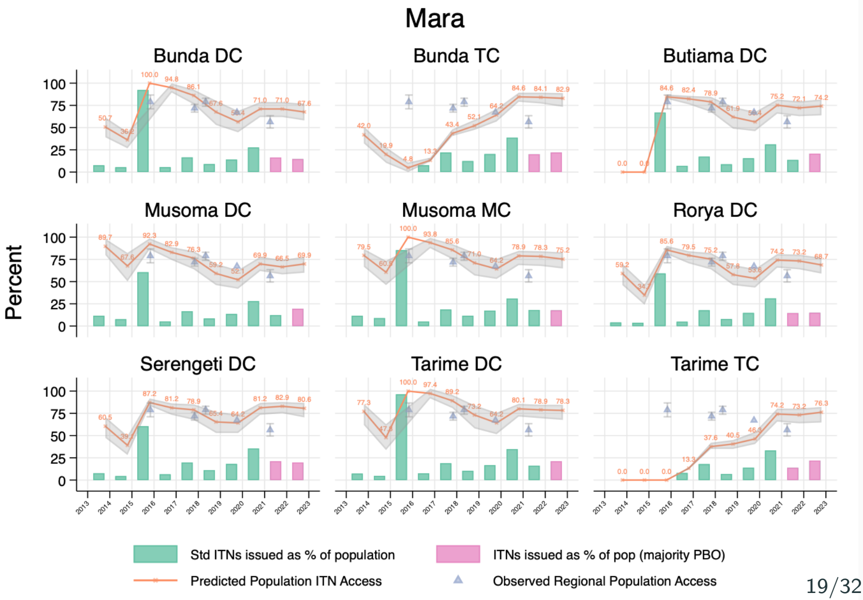
Mara
Bunda DC
Bunda TC
Butiama DC
Musoma DC
Musoma MC
Rorya DC
Serengeti DC
Tarime DC
Tarime TC
Percent
100
75
50
25
0
2013
2014
2015
2016
2017
2018
2019
2020
2021
2022
2023
Std ITNs issued as % of population
ITNs issued as % of pop (majority PBO)
Predicted Population ITN Access
Observed Regional Population Access

Morogoro

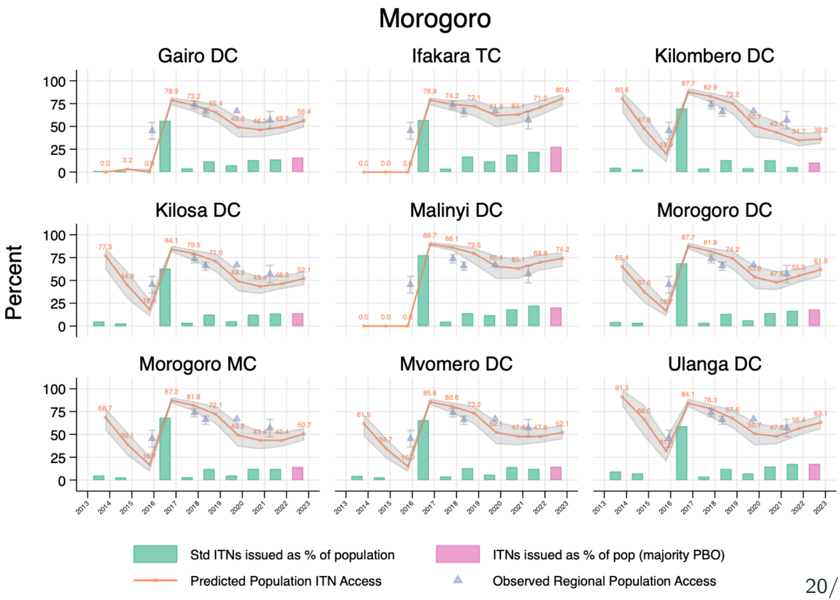
Morogoro
Gairo DC
Ifakara TC
Kilombero DC
Kilosa DC
Malinyi DC
Morogoro DC
Morogoro MC
Mvomero DC
Ulanga DC
Percent
0
25
50
75
100
2013
2014
2015
2016
2017
2018
2019
2020
2021
2022
2023
Std ITNs issued as % of population
ITNs issued as % of pop (majority PBO)
Predicted Population ITN Access
Observed Regional Population Access

Mtwara

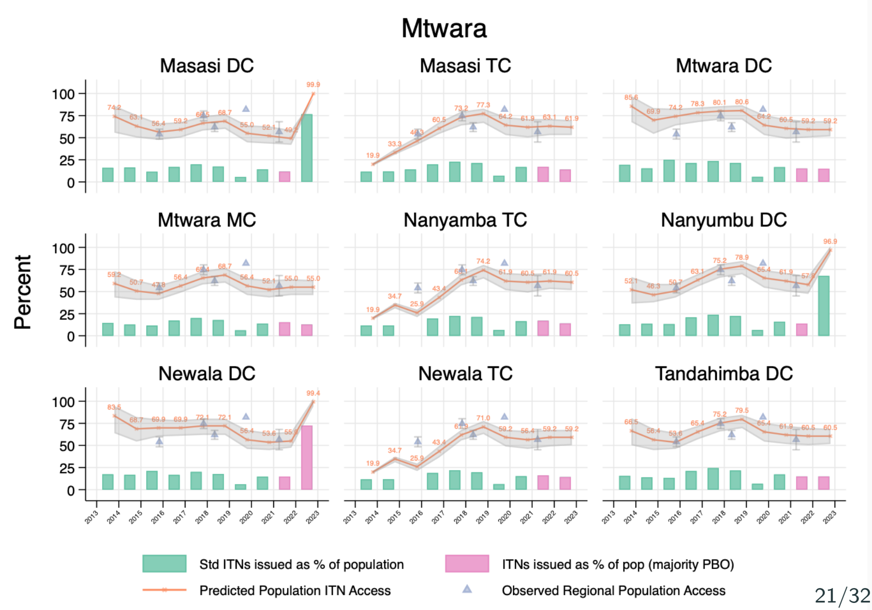
Mtwara
Masasi DC
Masasi TC
Mtwara DC
Mtwara MC
Nanyamba TC
Nanyumbu DC
Newala DC
Newala TC
Tandahimba DC
Percent
Std ITNs issued as % of population
ITNs issued as % of pop (majority PBO)
Predicted Population ITN Access
Observed Regional Population Access

Mwanza

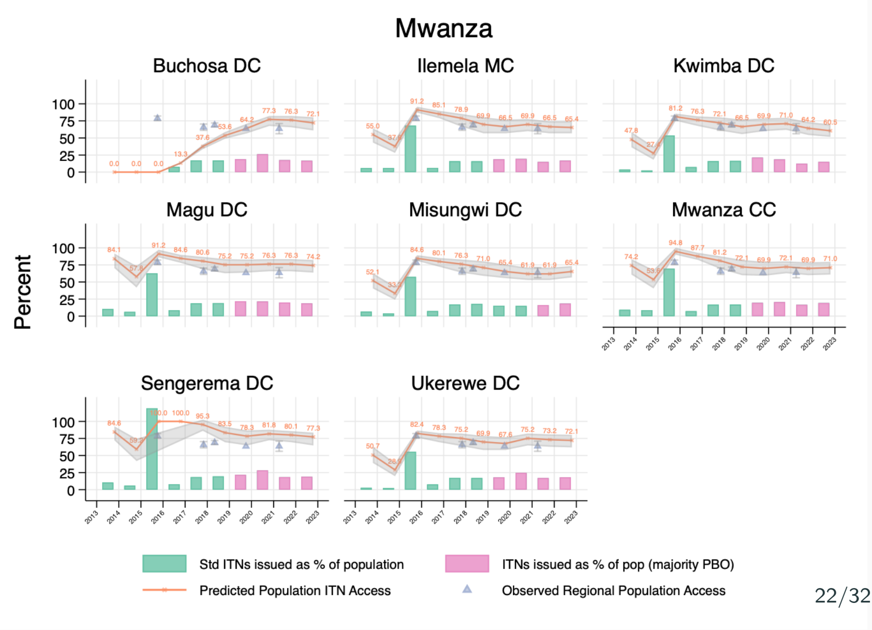

Mwanza
Buchosa DC
Ilemela MC
Kwimba DC
Magu DC
Misungwi DC
Mwanza CC
Sengerema DC
Ukerewe DC
Percent
Std ITNs issued as % of population
ITNs issued as % of pop (majority PBO)
Predicted Population ITN Access
Observed Regional Population Access

Pwani

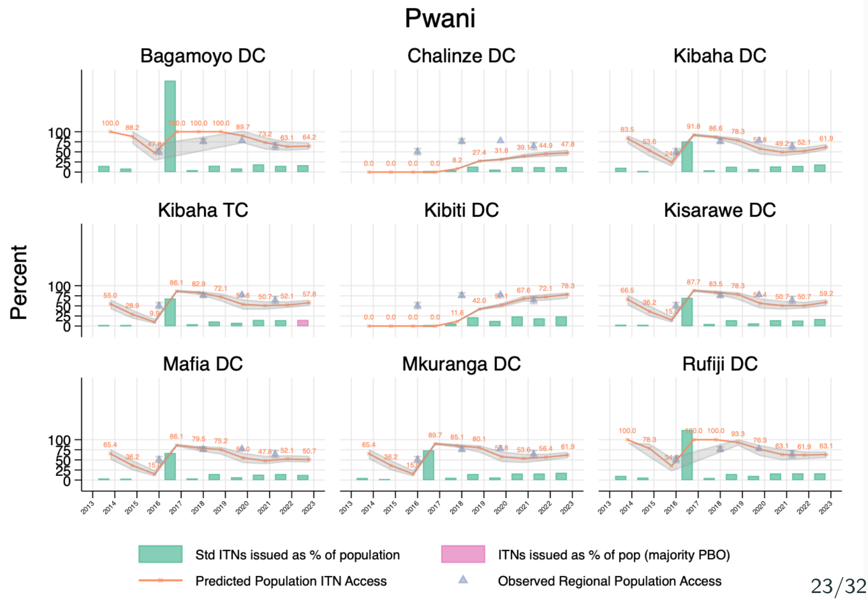

Pwani
Bagamoyo DC
Chalinze DC
Kibaha DC
Kibaha TC
Kibiti DC
Kisarawe DC
Mafia DC
Mkuranga DC
Rufiji DC
Percent
Std ITNs issued as % of population
ITNs issued as % of pop (majority PBO)
Predicted Population ITN Access
Observed Regional Population Access

Ruvuma

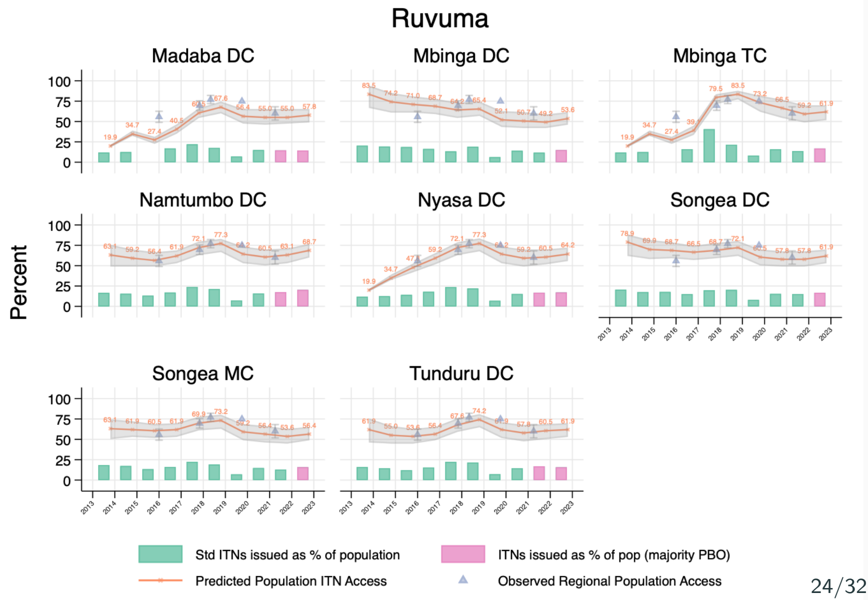
Ruvuma
Madaba DC
Mbinga DC
Mbinga TC
Namtumbo DC
Nyasa DC
Songea DC
Songea MC
Tunduru DC
Percent
Std ITNs issued as % of population
ITNs issued as % of pop (majority PBO)
Predicted Population ITN Access
Observed Regional Population Access

Shinyanga

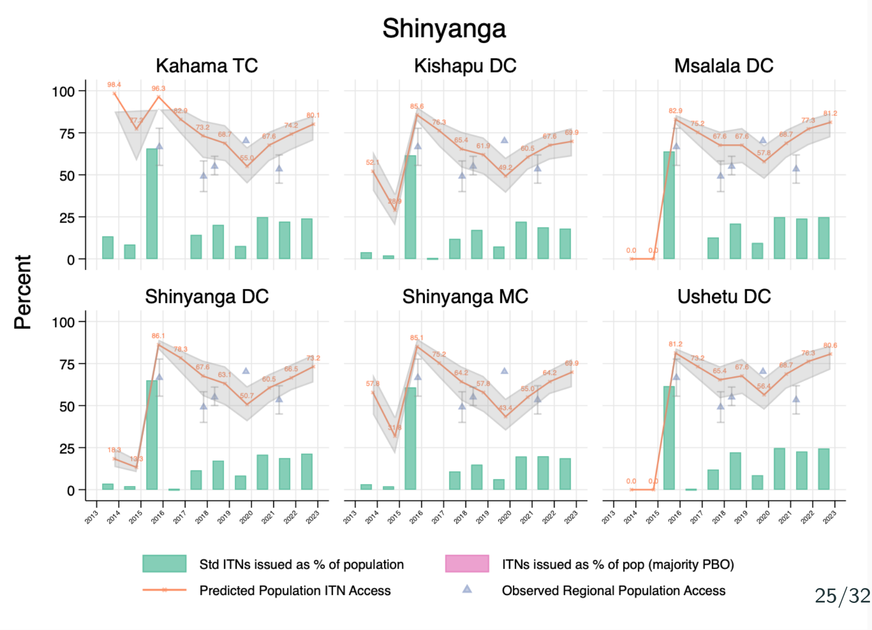
Shinyanga
Kahama TC
Kishapu DC
Msalala DC
Shinyanga DC
Shinyanga MC
Ushetu DC
Percent
Std ITNs issued as % of population
ITNs issued as % of pop (majority PBO)
Predicted Population ITN Access
Observed Regional Population Access

Simiyu

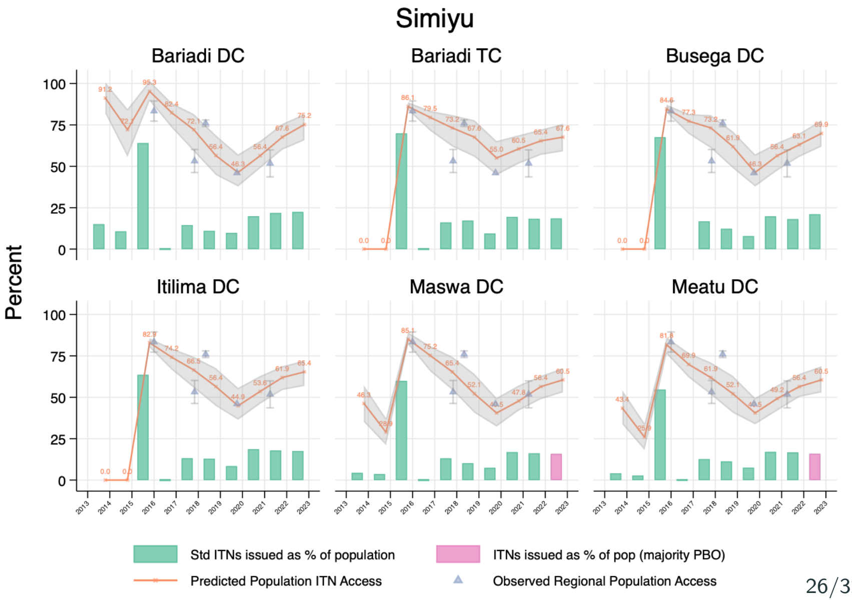

Simiyu
Bariadi DC
Bariadi TC
Busega DC
Itilima DC
Maswa DC
Meatu DC
Percent
100
75
50
25
0
2013
2014
2015
2016
2017
2018
2019
2020
2021
2022
2023
Std ITNs issued as % of population
ITNs issued as % of pop (majority PBO)
Predicted Population ITN Access
Observed Regional Population Access

Tabora

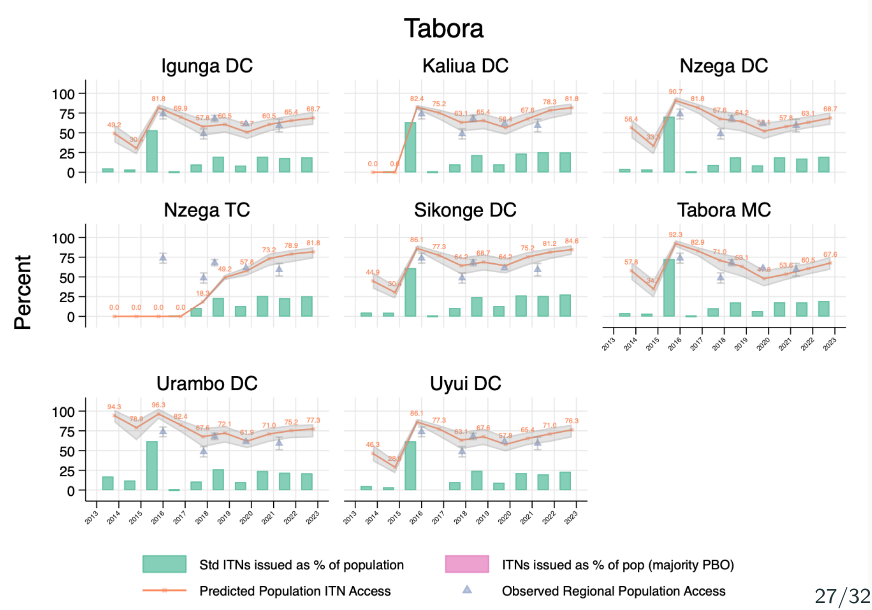
Tabora
Igunga DC
Kaliua DC
Nzega DC
Nzega TC
Sikonge DC
Tabora MC
Urambo DC
Uyui DC
Percent
Std ITNs issued as % of population
ITNs issued as % of pop (majority PBO)
Predicted Population ITN Access
Observed Regional Population Access

Zanzibar

Mjini Magharibi

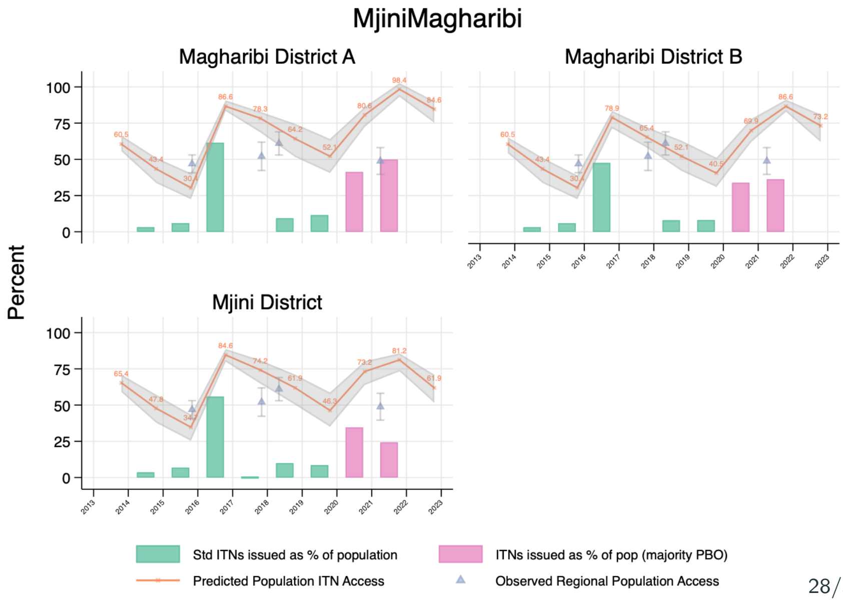

Kaskazini Unguja

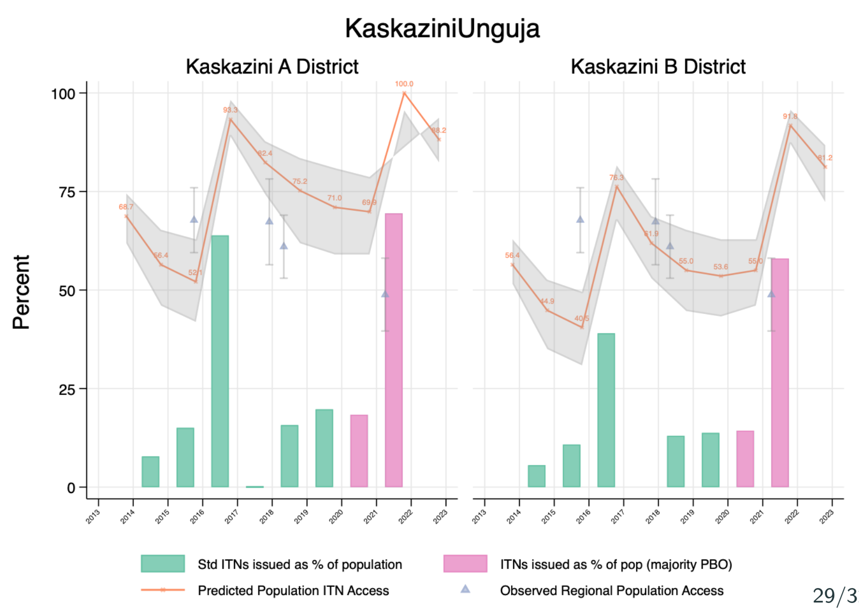

Kusini Unguja

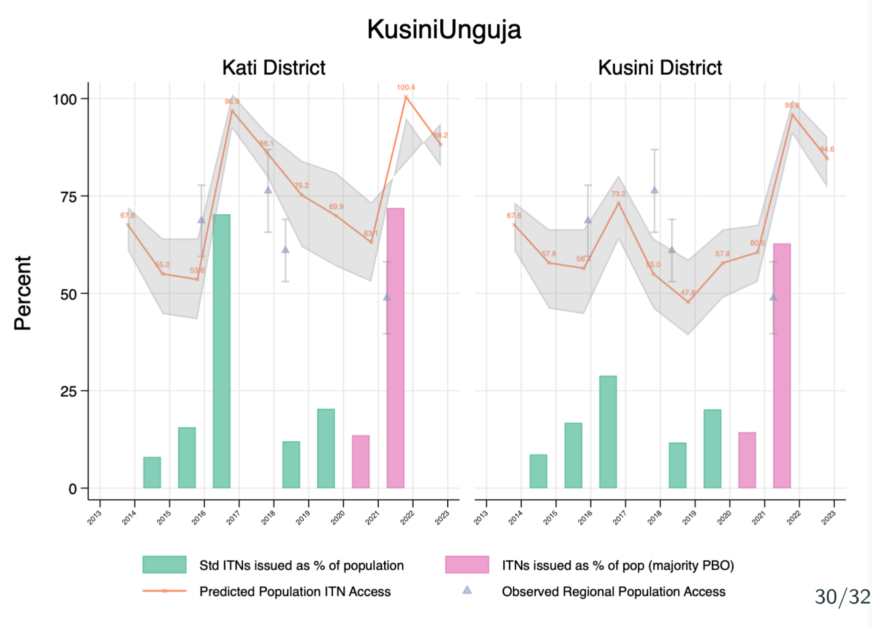

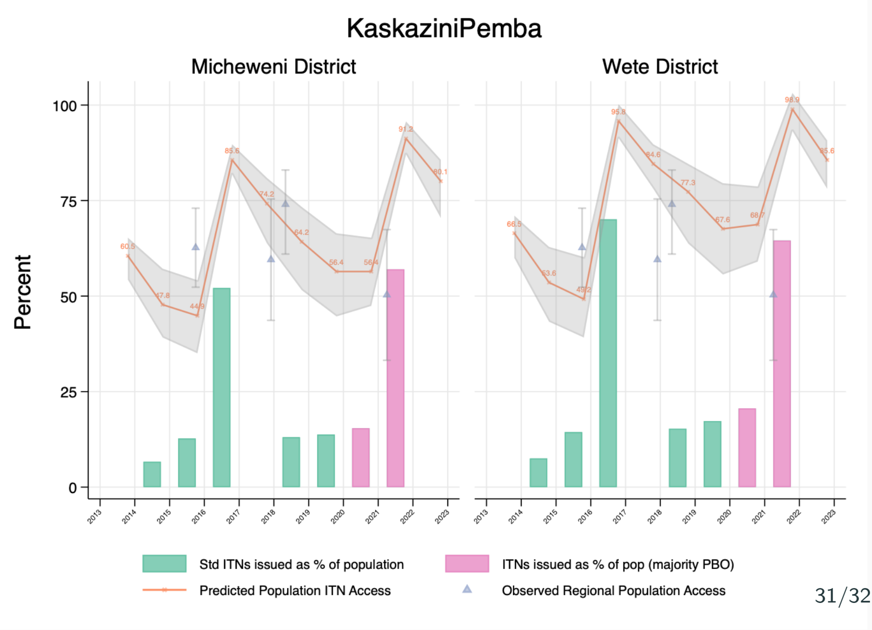
KaskaziniPemba
Micheweni District
Wete District
Percent
100
75
50
25
0
60.5
47.8
44.9
85.6
74.2
64.2
56.4
56.4
91.2
80.1
66.5
53.6
49.2
95.8
84.6
77.3
67.6
68.7
98.9
85.6
2013
2014
2015
2016
2017
2018
2019
2020
2021
2022
2023
Std ITNs issued as % of population
ITNs issued as % of pop (majority PBO)
Predicted Population ITN Access
Observed Regional Population Access

Kusini Pemba

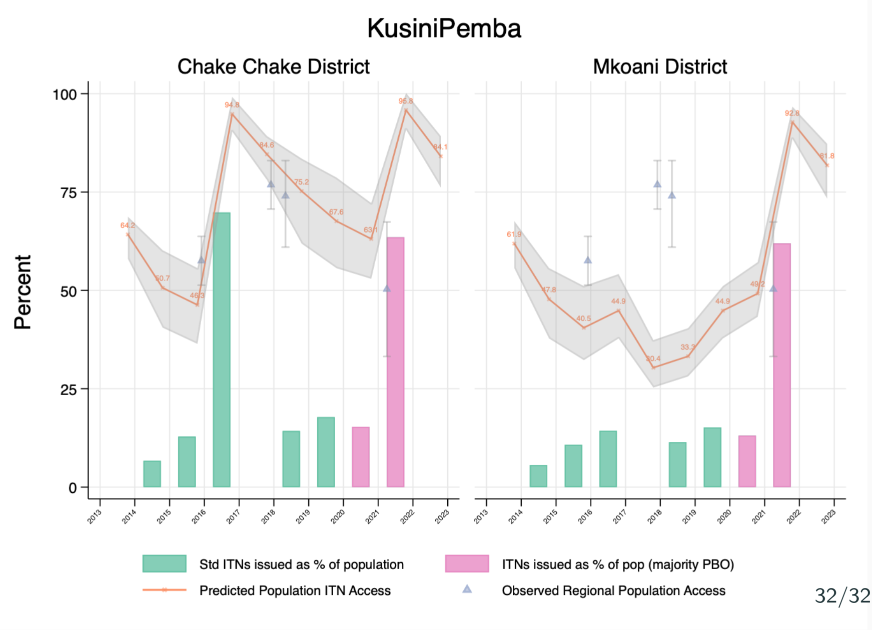

Supplement: Supplementary file 2 — Additional file 2. Graphs of estimated ITN access and ITNs issued from 2013–2022 for all councils in Tanzania. [file 12936_2022_4432_MOESM2_ESM.pdf]
