# Supplementary material for: Estimating population ITN access at council level in Tanzania
Source: Malar J. 2023 Jan 5;22:4. doi: 10.1186/s12936-022-04432-y (PMC9815063; doi:10.1186/s12936-022-04432-y)
Supplement: Supplementary file 3 — Additional file 3. Table of councils with incomplete or missing ITN or population data. [file 12936_2022_4432_MOESM3_ESM.docx]

Additional File 2: Councils with outlier estimates of ITN access due to classification anomalies – new council formation; change in boundaries; change in name; missing ITN delivery data; etc.

| Donor | Region | Council | Type of outlier | Notes |
| --- | --- | --- | --- | --- |
| PMI | Katavi | Mlele DC | MRC 2015 – NPP > 100% | Formed in 2012 out of part of Mpanda DC (Wikipedia entry). Split into Mlele and Mpimbwe in 2019 ([Katavi.go.tz/history](http://www.katavi.go.tz/history)) |
| PMI | Katavi | Mpimbwe DC | no data for 2015 MRC | Formed from Mlele DC in 2019 ([Katavi.go.tz/history](http://www.katavi.go.tz/history)) |
| PMI | Lindi | Mtama DC | n/a | Conflation with Lindi MC in several documents |
| PMI | Mara | Bunda TC | no data for 2015 MRC | Conflation with Bunda DC for MRC 2015 |
| PMI | Mara | Tarime DC | MRC 2015 – NPP > 100% | Conflation with TC in several documents |
| PMI | Mara | Tarime TC | no data for 2015 MRC | Established in 2013 ([www.tarimetc.go.tz/history](http://www.tarimetc.go.tz/history)). Conflation with DC in several documents |
| PMI | Mwanza | Buchosa DC | no data for 2015 MRC | Formed in Sept 2015 ([www.buchosadc.go.tz/history](http://www.buchosadc.go.tz/history)). Conflation with Sengerema prior to 2015 |
| PMI | Mwanza | Sengerema DC | MRC 2015 – NPP > 100% | Conflation with Buchosa prior to 2015 |
| PMI | Pwani | Bagamoyo DC | MRC 2015 – NPP > 100% | Divided in 2016 ([www.chalinzedc.go.tz/History](http://www.chalinzedc.go.tz/History)) |
| PMI | Pwani | Chalinze DC | no data for 2015 MRC | Formed from Bagamoyo DC in July 2016 ([www.chalinzedc.go.tz/History](http://www.chalinzedc.go.tz/History)) |
| PMI | Pwani | Kibiti DC | no data for 2015 MRC | Formed in 2015 from Rufiji DC |
| PMI | Pwani | Rufiji DC | MRC 2015 – NPP > 100% | Conflation with Kibiti DC prior to 2015 |
| PMI | Tabora | Nzega TC | no data for 2015 MRC | no data for 2015 MRC |
| Global Fund | Dodoma | Kondoa DC | MRC 2015 – NPP > 75% | Conflation with TC |
| Global Fund | Dodoma | Kondoa TC | no data for 2015 MRC | Conflation with DC |
| Global Fund | Manyara | Mbulu DC | MRC 2015 – NPP > 75% | Conflation with TC |
| Global Fund | Manyara | Mbulu TC | no data for 2015 MRC (only RCH data since 2014) | Conflation with DC in several documents |
| Global Fund | Singida | Itigi DC | no data for 2015 MRC | Established in Sept 2015 from Manyoni DC ([www.itigidc.go.tz/history](http://www.itigidc.go.tz/history)) |
| Global Fund | Singida | Manyoni DC | MRC 2015 – NPP > 100%; MRC 2020 – NPP>100% | Conflation with TC in several documents; includes Itigi DC prior to 2016 |
| Global Fund | Singida | Manyoni TC | Population, but no ITN data | Conflation with DC in several documents |
| Global Fund | Songwe | Songwe DC | no data for 2015 MRC; MRC 2020 – NPP>75% | Established in Sept 2015 ([www.songwedc.go.tz/history](http://www.songwedc.go.tz/history)) |
